# Combinatorial Effects of the Natural Products Arctigenin, Chlorogenic Acid, and Cinnamaldehyde Commit Oxidation Assassination on Breast Cancer Cells

**DOI:** 10.3390/antiox11030591

**Published:** 2022-03-20

**Authors:** Caroline Schuster, Nicholas Wolpert, Naima Moustaid-Moussa, Lauren S. Gollahon

**Affiliations:** 1 Department of Biological Sciences, Texas Tech University, Lubbock, TX 79409, USA; caroline.schuster@ttu.edu (C.S.); nicholas.wolpert@ttu.edu (N.W.); 2 Nutritional Sciences Department, Texas Tech University, Lubbock, TX 79409, USA; naima.moustaid-moussa@ttu.edu; 3 Obesity Research Institute, Texas Tech University, Lubbock, TX 79409, USA

**Keywords:** cinnamaldehyde, chlorogenic acid, arctigenin, breast cancer, cancer metabolism, mitochondria, ROS

## Abstract

Major obstacles in current breast cancer treatment efficacy include the ability of breast cancer cells to develop resistance to chemotherapeutic drugs and the off-target cytotoxicity of these drugs on normal cells, leading to debilitating side effects. One major difference between cancer and normal cells is their metabolism, as cancer cells acquire glycolytic and mitochondrial metabolism alterations throughout tumorigenesis. In this study, we sought to exploit this metabolic difference by investigating alternative breast cancer treatment options based on the application of phytochemicals. Herein, we investigated three phytochemicals, namely cinnamaldehyde (CA), chlorogenic acid (CGA), and arctigenin (Arc), regarding their anti-breast-cancer properties. These phytochemicals were administered alone or in combination to MCF-7, MDA-MB-231, and HCC1419 breast cancer or normal MCF-10A and MCF-12F breast cells. Overall, our results indicated that the combination treatments showed stronger inhibitory effects on breast cancer cells versus single treatments. However, only treatments with CA (35 μM), CGA (250 μg/mL), and the combination of CA + CGA (35 μM + 250 μg/mL) showed no significant cytotoxic effects on normal mammary epithelial cells, suggesting that Arc was the driver of normal cell cytotoxicity in all other treatments. CA + CGA and, to a lesser extent, CGA alone effectively induced breast cancer cell death accompanied by decreases in mitochondrial membrane potential, increased mitochondrial superoxide, reduced mitochondrial and glycolytic ATP production, and led to significant changes in cellular and mitochondrial morphology. Altogether, the combination of CA + CGA was determined as the best anti-breast-cancer treatment strategy due to its strong anti-breast-cancer effects without strong adverse effects on normal mammary epithelial cells. This study provides evidence that targeting the mitochondria may be an effective anticancer treatment, and that using phytochemicals or combinations thereof offers new approaches in treating breast cancer that significantly reduce off-target effects on normal cells.

## 1. Introduction

Breast cancer is the most prevalent cancer and the leading cause of cancer-related deaths in women worldwide. According to the International Agency for Research on Cancer (IARC), for the first time, breast cancer surpassed lung cancer as the most common cancer worldwide overall in 2020. It accounts for approximately 24.5% of the overall number of newly diagnosed cancer cases in women worldwide [1]. A major disadvantage of many of the currently used chemotherapeutics to treat breast cancer is normal cell cytotoxicity. Many of these target DNA (repair, replication, and division), which not only affects cancer cells, but also normal cells, leading to side effects that are systemic. In addition, a second major obstacle is the ability of breast cancer cells to develop resistance to chemotherapeutic drugs.

These hindrances highlight the need for different strategies to approach cancer treatment. One significant difference between cancer cells and normal cells is their metabolism. All stages of tumorigenesis are known to be affected by alterations in mitochondrial metabolism, including malignant transformation, resistance to cell death, tumor progression, proliferation, and metastasis [2,3,4]. Alterations to mitochondrial metabolism are often initially driven by mutations in nuclear or mitochondrial genes that lead to changes in metabolic pathways, the electron transport chain, and the maintenance of mitochondrial membrane potential [2,5,6]. This results in increased reactive oxygen species (ROS) production [7,8], changes in the mitochondrial fission and fusion rates [9], evasion of cell death [3,4,10], and increased oncometabolite production induced by mutations in mitochondrial enzymes [3,4,11,12,13], all of which further drive oncogenesis. Therefore, due to the inherent differences in metabolism between normal and cancer cells, targeting metabolism has emerged as an attractive target of cancer treatment to selectively induce cancer cell death [14]. One challenge of targeting cancer cell metabolism is the metabolic adaptability of cancer cells, allowing them to evade treatments targeting them. Studies have demonstrated that some cancer cells have the ability to switch between oxidative phosphorylation and glycolysis as their ATP supplier when their main ATP-producing mechanism is targeted by treatment [4,15,16].

Another potential cancer treatment approach that is emerging is combining several phytochemicals with anti-cancer potential. This approach is in direct contrast to more traditional methods of drug development that primarily use single compounds. Several studies have shown that combining several plant isolates, rather than single active components isolated from plants, may increase anti-cancer potential [17,18,19]. This strategy may lead to synergistic effects by increasing the number of pathways and systems that can be targeted at once, potentially decreasing resistance to treatment [20].

In this study, three phytochemicals were selected for their potential as effective anti-breast-cancer agents for combination treatments, namely cinnamaldehyde (CA), chlorogenic acid (CGA), and arctigenin (Arc). CA is most commonly found in the bark of cinnamon tree species, including *Cinnamomum cassia*, *Cinnamomum camphor*, and *Cinnamomum zeylanicum*, and is a widely used spice. CGA is one of the major polyphenols primarily found in coffee beans, but it is also present in many foods, such as fruits and vegetables. Arc is primarily found in the Greater Burdock plant (*Arctium lappa*), better known as Greater Burdock. All three are known antioxidants and are beneficial to human health [21,22,23,24,25,26,27,28,29,30]. Furthermore, CA, CGA, and Arc have [31] previously been shown to have anti-breast-cancer potential [31,32,33,34,35,36,37,38,39,40,41,42,43,44,45,46,47,48,49,50,51,52,53,54] and to induce apoptosis through the mitochondrial-dependent pathway to decrease the mitochondrial membrane potential and increase reactive oxygen species (ROS) in several cancer cells [39,55,56,57,58,59,60,61,62,63,64,65,66,67,68,69,70,71].

CA, CGA, and Arc have each been shown to have synergistic anticancer effects when combined with certain compounds. For example, they have been shown to have the ability to chemosensitize cancer cells to various chemotherapeutics [31,53,72,73,74,75,76,77,78,79,80,81]. Moreover, several studies have demonstrated their ability to synergize with other plant-derived and natural compounds, where they potentiated anticancer effects [52,82,83,84,85]. Several studies have investigated the cytotoxic effects of CA, CGA, and Arc, and many found little to no cytotoxic activity of these agents on normal cells compared to cancer cells [34,62,63,68,86,87,88,89,90,91,92,93,94].

Herein, we investigated CA, CGA, and Arc alone and in combination in breast cancer as well as normal cells, in regard to their effects on cell growth, cell death, metabolism, and ROS production.

## 2. Materials and Methods

### 2.1. Cell Lines and Cell Culture

The breast cancer cell lines used were MCF-7, MDA-MB-231, and HCC1419. MCF-7 breast cancer cells are ER- and PR-positive and HER2-negative, whereas MDA-MB-231 cells are triple-negative [95]. HCC1419 cells do not express ER or PR and overexpress HER2 [96]. The MCF-7, MDA-MB-231, and HCC1419 breast cancer cell lines were cultured in high-glucose Dulbecco’s Modified Eagle’s Medium (DMEM; Gibco, Waltham, MA, USA) supplemented with 10% fetal bovine serum (FBS; ATCC, Rockville, MD, USA) and 1% penicillin/streptomycin. MCF-10A and MCF-12F mammary epithelial cells were purchased from ATCC and cultured in DMEM/Ham’s F-12 (1:1) (Gibco, Waltham, MA, USA) supplemented with 5% horse serum, 20 ng/mL epidermal growth factor (EGF; Gibco, Waltham, MA, USA), 0.5 µg/mL hydrocortisone (Sigma–Aldrich, Saint Louis, MO, USA), 100 ng/mL cholera toxin (Sigma–Aldrich, Saint Louis, MO, USA), 10 µg/mL insulin (Sigma–Aldrich, Saint Louis, MO, USA), and 1% penicillin/streptomycin. All cells were cultured at 37 °C and 5% CO_2_.

### 2.2. Cell Treatments

Cinnamaldehyde (CA) was obtained from Sigma–Aldrich (Sigma–Aldrich, Saint Louis, MO, USA) (≥95% purity) in liquid form. CA was diluted in dimethyl sulfoxide (DMSO; ATCC, Rockville, MD, USA) to a stock concentration of 79.449 mM. Chlorogenic acid (CGA) was purchased from Sigma–Aldrich (Sigma–Aldrich, Saint Louis, MO, USA) (≥95% purity) in powder form and was dissolved in DMSO to a stock concentration of 100 g/L. Arctigenin (Arc) was obtained from Tocris Bioscience (Bristol, UK) (98.6% purity) in powder form. It was dissolved in DMSO to a stock concentration of 20 mM. Stock solutions were stored at −20 °C for further use. At the time of cell treatment, stock solutions were added to appropriate cell media and then filtered through 0.22 μm-pore-size syringe filters (MilliporeSigma/Merck KGaA, Darmstadt, Germany).

### 2.3. Cell Growth Curves

MCF-7 (HTB-22 from ATCC, Manassas, VA, USA), MDA-MB-231 (HTB-26 from ATCC, Manassas, VA, USA), MCF-10A (CRL-10317 from ATCC, Manassas, VA, USA), and MCF-12F (CRL-10783 from ATCC, Manassas, VA, USA) cells were seeded at 20,000 cells/well into 48-well plates (ThermoFisher Scientific, Waltham, MA, USA), allowed to grow for 24 h, and then treated for 7 days. HCC1419 (CRL-2326 from ATCC, Manassas, VA, USA) cells were seeded at 10,000 cells/well and treated for 6 days. Each day, cells were washed with phosphate-buffered saline (PBS) and then harvested using 0.25% trypsin-EDTA (Gibco, Waltham, MA, USA) for MCF-7, MDA-MB-231, HCC1419, and MCF-10A, and 0.05% trypsin-EDTA for MCF-12F cells. PBS and trypsinized cells were collected and the number of cells in each well was counted using a Scepter™ 2.0 Cell Counter. The IC_50_ concentrations of CA, CGA, and Arc were established by a 50% cell growth reduction of each single treatment group compared to the untreated group over a period of 7 days (or 6 days for HCC1419 cells). The IC_50_ concentrations of CA, CGA, and Arc were used to create the combination treatments CA + CGA, CA + Arc, CGA + Arc, and CA + CGA + Arc. DMSO (ATCC, Rockville, MD, USA) was used as the vehicle control at a concentration equivalent to the amount used in the CA + CGA + Arc treatment group, as this group contained the highest amount of DMSO.

### 2.4. Cell Death Assay

MCF-7, MDA-MB-231, HCC1419, MCF-10A, and MCF-12F cells were seeded at 40,000 cells/well into 24-well plates and allowed to grow for 24 h. The cells were then left untreated or treated with single and combination treatments of CA, CGA, and Arc for 4 days. The cells were stained with MitoTracker^®^ Red CMXRos (ThermoFisher Scientific, Waltham, MA, USA) and Annexin V-FITC (eBioscience^TM^, Invitrogen, Waltham, MA, USA). Twenty-four hours prior to staining treatment, the cells were treated with 10 μM Sutent (Pfizer, New York, NY, USA) as a control for the Annexin V-FITC-positive signal and a 30 min treatment with 0.1% Triton X-100 (Sigma–Aldrich, Saint Louis, MO, USA) was used as the MitoTracker^®^ Red CMXRos and Annexin V-FITC negative control. Cells were stained with MitoTracker^®^ Red CMXRos by directly adding the dye to the wells to make a final concentration of 0.04 μM. The cells were then incubated for 30 min at 37 °C in 5% CO_2_. Afterward, the cells were washed with PBS and then harvested using 0.25% trypsin-EDTA (Gibco, Waltham, MA, USA) for MCF-7, MDA-MB-23, HCC1419, and MCF-10A and 0.05% trypsin-EDTA for MCF-12F cells. The cell medium, PBS, and trypsinized cells were collected and cells were spun down and resuspended in 1× annexin binding buffer (eBioscience^TM^/Invitrogen, Waltham, MA, USA). Subsequently, the cells were incubated with Annexin V-FITC for 15 min at room temperature. Data acquisition was performed by flow cytometry using an Attune NxT Flow Cytometer (Invitrogen, Waltham, MA, USA). The FSC-H and SSC-H channels were used for cell morphology and size. The BL1-H channel was used to quantify the fluorescence associated with Annexin V-FITC and the YL1-H channel for MitoTracker^®^ Red CMXRos. The excitation of Annexin V-FITC positive cells was performed with a 488 nm blue laser and emission was recorded at 530/30 nm. MitoTracker^®^ Red CMXRos positive cells were excited with a 561 nm yellow laser and recorded at 585/16 nm. See Table 1 for channel voltages optimized for each cell line.

The threshold was set on FSC to 50,000 for MCF-7 and MDA-MB-231 and 100,000 for HCC1419, MCF-10A, and MCF-12F to eliminate events that are smaller than the respective threshold channel numbers. Doublets were excluded by plotting FSC-A against FSC-H. For data analysis, Annexin V-FITC fluorescence was plotted against MitoTracker^®^ Red CMXRos fluorescence. Untreated, unstained samples, MitoTracker^®^ Red CMXRos single stained samples, and 10 μM Sutent-treated Annexin V-FITC single stained samples were used for compensation. Quadrant placement was kept equal among the biological and experimental replicates. The percentages of cells in each quadrant were recorded and analyzed with the FlowJo software (version 10.7.1).

### 2.5. 40× Fluorescence Microscopy and Phase–Contrast Imaging

MCF-7 and MCF-10A cells were seeded at a density of 20,000 cells/well into 48-well plates and allowed to grow for 24 h. The cells were then treated with single and combination treatments of CA, CGA, and Arc, or fresh medium for the untreated group, for 6 h and 24 h. After 6 h or 24 h, the cells were incubated with MitoTracker^®^ Green FM (Sigma–Aldrich, Saint Louis, MO, USA) to a final concentration of 150 μM of MitoTracker^®^ Green FM for 30 min and Hoechst 33342 to a final concentration of 1 μg/mL for 15 min at 37 °C by direct addition to cell medium and subsequent mixing. After incubation, the cells were washed once with warm PBS and FluoroBrite^TM^ DMEM medium (Sigma–Aldrich, Saint Louis, MO, USA) was added for cell imaging. The cells were imaged using the EVOS™ M7000 Imaging System (Invitrogen, Waltham, MA, USA). The cells were visualized with a 40× objective and DAPI (Ex: 357/44; Em: 447/60) and GFP (Ex: 482/25; Em: 524/24) filter cubes. All settings were kept equal between samples of the same cell lines and between biological replicates. The images were processed and analyzed using FIJI [97]. All brightness/contrast adjustments were kept equal between all samples of the same cell lines. The MCF-7 cell area was determined by manually drawing around individual cells in phase–contrast images and measuring the area in FIJI.

### 2.6. 100× Fluorescence Microscopy Imaging

MCF-7 cells were seeded at a density of 80,000 cells/well on coverslips in a 12-well plate and allowed to grow for 24 h. The cells were then treated with CGA, Arc, or fresh medium for the untreated group for 24 h. The cells were then incubated with 150 μM MitoTracker^®^ Green FM (ThermoFisher Scientific, Waltham, MA, USA) for 30 min at 37 °C. After staining, the cells were fixed in 4% paraformaldehyde in PBS for 15 min. The cells were then washed with warm HBSS/Ca/Mg and coverslips with cells were mounted with DAPI/Antifade (Sigma–Aldrich, Saint Louis, MO, USA). The cells were visualized with the 100× objective and DAPI (Ex: 365/10; Em: 420LP) and GFP (Ex: 480/20; Em: 510LP) filter cubes using the Olympus BX51 microscope (Olympus, Center Valley, PA, USA). All settings were kept equal between the samples. The images were processed and analyzed using FIJI [97]. All brightness/contrast adjustments were kept equal between all samples.

### 2.7. Mitochondrial Superoxide Production Assay

MCF-7 and MCF-10A cells were seeded at 40,000 cells/well into 24-well plates and allowed to grow for 24 h. MitoSOX™ Red (ThermoFisher Scientific, Waltham, MA, USA) staining solution was prepared on the day of the experiment by dissolving the vial contents into 13 μL of DMSO for the stock solution. To produce the working solution, MitoSOX™ Red was diluted in HBSS/Ca/Mg (Gibco, Waltham, MA, USA) for a 5 μM solution. The cells were treated with single and combination treatments of CA, CGA, and Arc, or fresh medium for the untreated group, for 2 h. The medium was then removed, and the cells were washed with warm PBS. The cells were stained with 5 μM MitoSOX™ Red for 10 min at 37 °C. Afterward, the cells were washed twice with warm PBS. For cell imaging, warm FluoroBrite^TM^ DMEM (Gibco, Waltham, MA, USA), free of Phenol Red and L-Glutamine, was added to the cells. The cells were visualized with 10× and 40× objectives and the RFP filter cube (Ex: 531/40; Em: 593/40) using the EVOS™ M7000 Imaging System (ThermoFisher Scientific, Waltham, MA, USA). All settings were kept equal between samples of the same cell lines and between experimental replicates. The images were processed and analyzed using FIJI [97]. For proper thresholding, the adaptive thresholding plugin by Qingzong Tseng, Ph.D., was used. All brightness/contrast adjustments were kept equal between all samples of the same cell line. Mitochondrial superoxide production was determined by calculating the integrated densities in FIJI.

### 2.8. ATP Production Rate Assay

MCF-7 cells were seeded at a density of 10,000 cells/well into a V7 PS cell culture microplate (24 wells; Agilent Technologies, Santa Clara, CA, USA) and allowed to grow for 24 h. MCF-7 cells were then treated with either fresh medium for the untreated control group, CA, CGA, Arc, CA + CGA, or CA + CGA + Arc for 48 h. The standard protocol for the Agilent Seahorse XF Real-Time ATP Rate Assay, as described in the Agilent Seahorse XF Real-Time ATP Rate Assay Kit User Guide (Kit 103592-100, Agilent Technologies, Santa Clara, CA, USA), was followed. The day before the assay, the sensor cartridge was placed in a 37 °C and non-CO_2_ incubator to hydrate overnight. On the day of the assay, the cells were washed once with 37 °C pre-warmed Seahorse XF DMEM Medium, pH 7.4, supplemented with 10 mM of XF glucose, 1 mM of XF pyruvate, and 2 mM of XF glutamine. After washing, fresh Seahorse XF DMEM Medium with supplements was added to the microplate wells and the cells were incubated at 37 °C in a non-CO_2_ incubator for 45 min. Meanwhile, oligomycin and rotenone + antimycin A injection compounds were prepared and loaded into the sensor cartridge to achieve final well concentrations of 1.5 µM and 0.5 µM, respectively. The sensor cartridge was then placed in the Seahorse XFe24 Analyzer. The medium previously added to cells was removed and fresh Seahorse XF DMEM Medium with supplements was added to the cells, and the microplate was placed in the Seahorse XFe24 Analyzer. During the assay, the Seahorse XFe24 Analyzer measured the oxygen consumption rate (OCR) and extracellular acidification rate (ECAR) at baseline, after oligomycin injection, and after rotenone + antimycin A injection. The total, mitochondrial, and glycolytic ATP production rates were calculated using the Seahorse Wave software (version 2.6.1) and Seahorse XF Real-Time ATP Rate Assay Report Generator (version 4.0.17). The results were normalized by quantifying the total proteins in each well using the Pierce™ BCA Protein Assay Kit (ThermoFisher Scientific, Waltham, MA, USA) and a LUMIstar Omega plate reader.

### 2.9. Statistical Analyses

All statistical analyses were performed in either R or SPSS. In the cell growth experiments, 3 biological replicates were used in each treatment group (sample size per treatment group: *n* = 3). For each day, a separate one-way ANOVA was performed, where ‘Treatment’ was used as the independent variable and ‘Number of Cells’ or ‘Ratio of Treated/Untreated’ was used as the dependent variable. In the cell death assays, for each cell line, the experiments were repeated thrice and each treatment group had 3 biological replicates. For each treatment group, the 3 biological replicates were averaged and used as one individual data point, resulting in a sample size of *n* = 3 per treatment group overall. ‘Treatment’ was used as the independent variable and ‘Percent of Live Cells’ as the dependent variable in the one-way ANOVA. The statistical significance in the cell area from the phase–contrast images between treatment groups was established by averaging the cell areas from 10 images per biological replicate in each treatment group, with 3 biological replicates each, resulting in an overall sample size of *n* = 3 per treatment group. One-way ANOVAs were performed with ‘Treatment’ as the independent variable and ‘Cell Area’ as the dependent variable. In the mitochondrial superoxide production assay, experiments were repeated thrice per cell line. The integrated density of each treatment group for each repeated experiment was established by averaging the integrated densities from 8–10 images per treatment group, resulting in an overall sample size of *n* = 3 per treatment group. One-way ANOVAs were performed with ‘Treatment’ as the independent variable and ‘Integrated Density’ as the dependent variable. For the ATP production rate assay, experiments were repeated 4 times and each treatment group had 3–5 biological replicates. The overall sample size per treatment group for the ANOVA was *n* = 4, as the biological replicates of each repeated experiment were averaged into one data point. Separate one-way ANOVAs were performed for the total ATP production rate, mitochondrial ATP production rate, and glycolytic ATP production rate. In each, ‘Treatment’ was used as the independent variable, while ‘Total ATP Production Rate’, ‘Mitochondrial ATP Production Rate’, and ‘Glycolytic ATP Production Rate’ were used as the dependent variables. All one-way ANOVAs were followed by a post hoc Tukey HSD test if the ANOVA was significant. For all statistical analyses, *p*-values of ≤ 0.05 were considered statistically significant.

## 3. Results

### 3.1. IC_50_ Concentrations of CA, CGA, and Arc Treatments of Breast Cancer Cells

**Figure 1:**
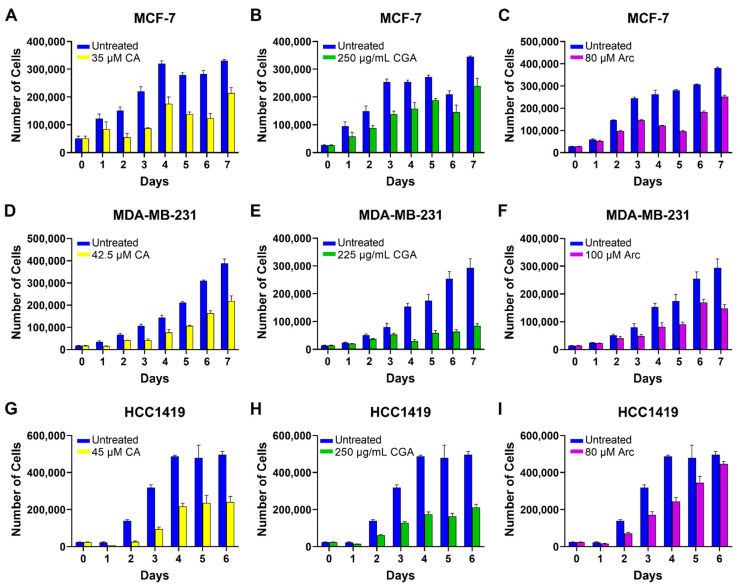
IC_50_ concentrations of CA, CGA, and Arc-treated breast cancer cells over time. Cell growth was determined by counting the number of cells in each group on each day. IC_50_ concentrations of (**A**) CA-treated MCF-7 cells, (**B**) CGA-treated MCF-7 cells, (**C**) Arc-treated MCF-7 cells, (**D**) CA-treated MDA-MB-231 cells, (**E**) CGA-treated MDA-MB-231 cells, (**F**) Arc-treated MDA-MB-231 cells, (**G**) CA-treated HCC1419 cells, (**H**) CGA-treated HCC1419 cells, and (**I**) Arc-treated HCC1419 cells. Each bar represents the mean ± SEM of three biological replicates (*n* = 3).

The IC_50_ concentrations of CA, CGA, and Arc on breast cancer cell lines of different molecular subtypes were determined by using MCF-7, MDA-MB-231, and HCC1419 breast cancer cells. The cell growth curves were established over a period of 7 days (6 days for HCC1419 due to the maximum confluency on day 6). Based on the concentration values of previous publications [31,33,34,39,44,46,47,48,49,50], different concentrations were tested (Figures S1–S3) and IC_50_ concentrations were determined. In MCF-7 breast cancer cells, the IC_50_ concentrations for CA, CGA, and Arc treatment were found to be 35 µM, 250 µg/mL, and 80 µM, respectively (Figure 1A–C). The IC_50_ concentrations for MDA-MB-231 breast cancer cells were 42.5 µM CA, 225 µg/mL CGA, and 100 µM Arc (Figure 1D–F). In the HCC1419 breast cancer cells, the IC_50_ concentration for CA treatment was 45 µM, 250 µg/mL for CGA treatment, and 80 µM for Arc treatment (Figure 1G–I).

### 3.2. Combination Treatments of CA, CGA, and Arc Are Effective in Reducing Breast Cancer Cell Growth

**Figure 2:**
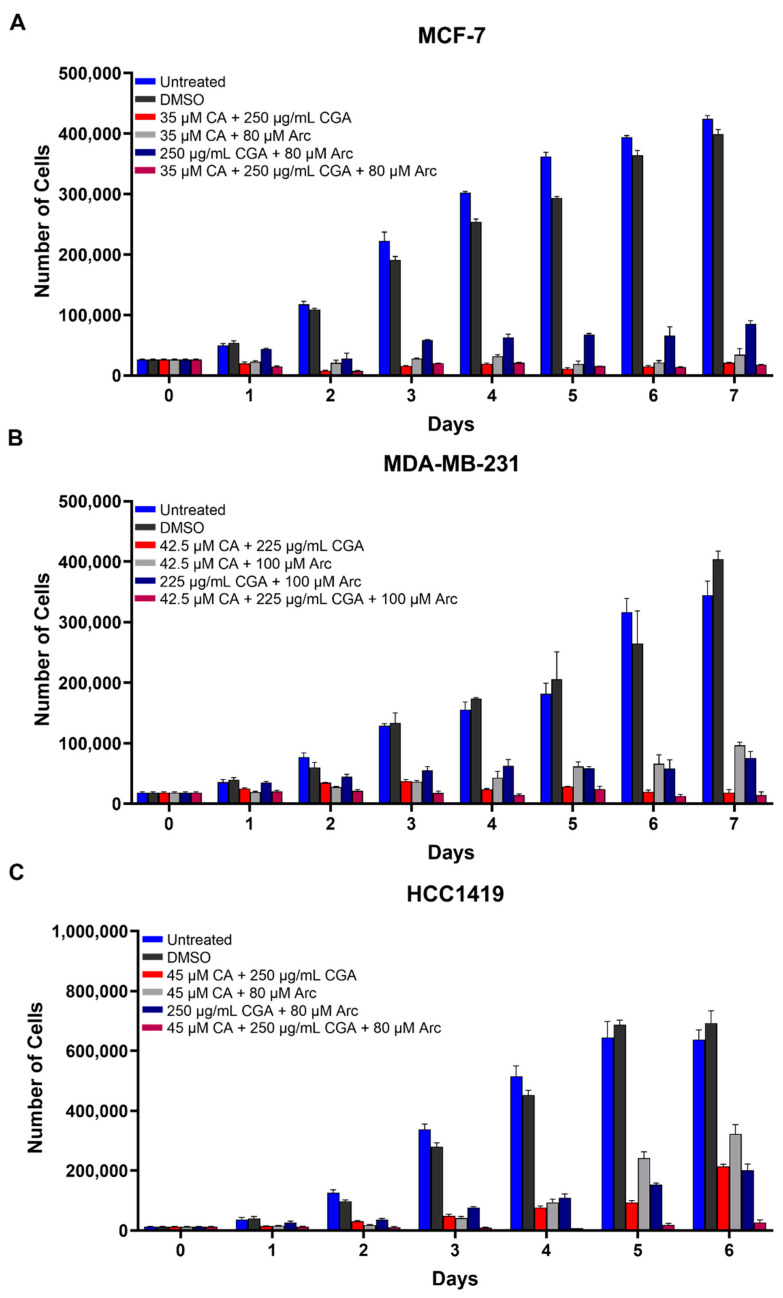
Breast cancer cell growth curves treated with combinations of CA, CGA, and Arc in (**A**) MCF-7, (**B**) MDA-MB-231, and (**C**) HCC1419 cells. Cell growth was determined by counting the number of cells in each group on each day for 6/7 days. Each bar represents the mean ± SEM of three biological replicates (*n* = 3). Groups were compared using a one-way ANOVA. The results of the statistical analysis are summarized in Tables S1–S3. Cell growth differences between groups were considered significant at *p* ≤ 0.05.

Furthermore, the effectiveness of combination treatments on breast cancer cell growth reduction was examined and were compared to the single treatments. Combination treatments were created by using the respective IC_50_ concentrations of CA, CGA, and Arc and combining them in culture media. The combination treatments were as follows: CA + CGA, CA + Arc, CGA + Arc, and CA + CGA + Arc. Overall, the combination treatments were shown to be very potent in reducing breast cancer cell growth. In the MCF-7 breast cancer cells, all combination treatments led to a significant reduction in cell growth compared to the untreated and DMSO-treated cells with the exception of CGA + Arc treatment on Day 1. CA + CGA, CA + Arc, and CA + CGA + Arc combination treatments were more effective than the CGA + Arc combination on most days in this cell line (Figure 2A and Table S1).

Similarly, combination treatments were very effective in reducing MDA-MB-231 cell growth compared to untreated and DMSO-treated cells. The CA + CGA and CA + CGA + Arc combination groups resulted in the highest cell reductions and there was no statistically significant difference between them. It is of note, however, that CGA + Arc was not statistically significant from the untreated group on Day 1, but it significantly reduced cell growth compared to the DMSO group. The CA + Arc group was similarly as effective as CA + CGA and CA + CGA + Arc to Day 4, after which, while significantly different from the controls, it performed statistically worse than the other combinations (Figure 2B and Table S2).

Combination treatments also significantly reduced HCC1419 cell growth compared to untreated and DMSO-treated cells, with the exception of the CGA + Arc treatment on Day 1. CA + CGA + Arc treatment was the most potent group, but was only significantly more effective than the CA + CGA group on Day 7. It was found to be more potent than the CA + Arc group on Days 4–6 and the CGA + Arc group on Days 2–6. There was no significant difference between the double treatment groups, except on Day 5, where CA + CGA was more potent than CA + Arc (Figure 2C and Table S3).

### 3.3. Combination Treatments of CA, CGA, and Arc Are More Effective in Reducing Breast Cancer Cell Growth than Single Treatments

**Figure 3:**
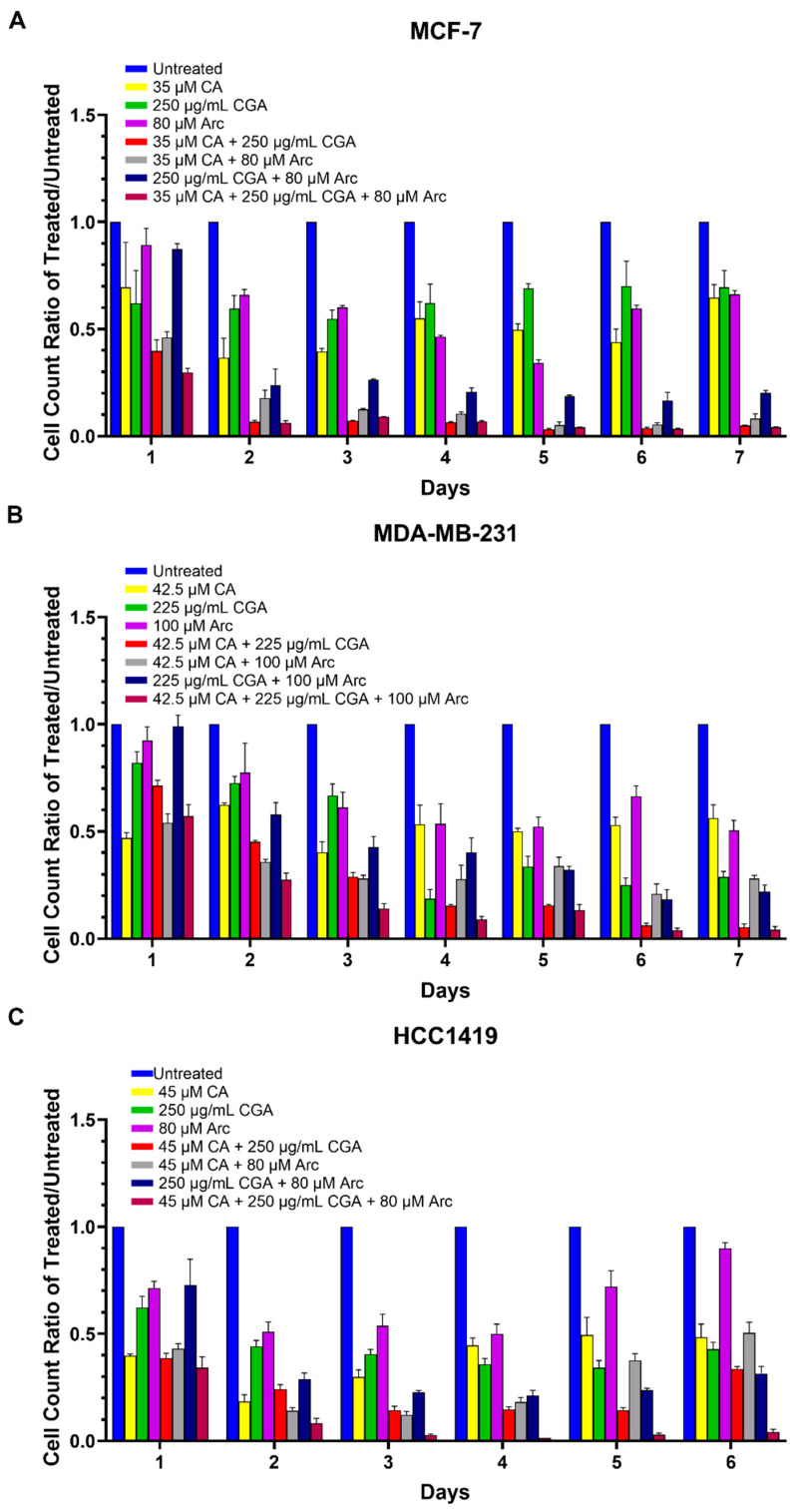
Ratios of cell numbers of each treatment group to the untreated group in (**A**) MC7-7, (**B**) MDA-MB-231, and (**C**) HCC1419 breast cancer cells. Each bar represents the mean ± SEM of three biological replicates (*n* = 3). The treatment groups were compared using a one-way ANOVA. The results of the statistical analysis are summarized in Tables S4–S6. Cell growth differences between treatment groups were considered significant at *p* ≤ 0.05.

The ratios of the cell numbers of each treatment group to the untreated group were used to determine whether the combination treatments were more effective in reducing breast cancer cell growth compared to the single treatments. Overall, the combination treatments were found to be more effective in reducing breast cancer cell growth than the CA, CGA, and Arc treatments individually. The CA treatment in MCF-7 cells was significantly less effective than the CA + CGA and CA + CGA + Arc groups on Days 2–7 and less potent than the CA + Arc and CGA + Arc groups on Days 3–7. Except for Day 1, all combination groups were more effective in reducing MCF-7 breast cancer cell growth compared to CGA or Arc alone, whereas CA + CGA + Arc was more effective than CGA and Arc, even on Day 1 (Figure 3A and Table S4).

In the MDA-MB-231 cells, the CA + CGA + Arc combination was significantly more successful in reducing breast cancer cell growth than any single treatment group on all days, except when compared to CA on Day 1. CA was less effective than CA + CGA and CGA + Arc on Days 1, 5, 6, and 7, and CA + Arc on Days 5–7. CGA was also less potent than CA + CGA on Days 3, 5, 6, and 7, less than CA + Arc on Days 1–4, and less than CGA + Arc on Day 3. Arc was less effective than CA + CGA on Days 2–7, CA + Arc on days 1–7, and CGA + Arc on Days 5–7 (Figure 3B and Table S5).

In HCC1419 breast cancer cells, the treatment of CA had lower inhibitory effects on cell growth compared to CA + CGA on Days 3 and 5, CA + Arc on Day 3, CGA + Arc on Days 1 and 5, and CA + CGA + Arc on Days 3, 5, and 6. CGA-treated HCC1419 cells showed lower cell growth reduction than cells treated with CA + CGA, CA + Arc, and CGA + Arc on Days 2–4, and with CA + CGA + Arc on Days 1–6. Arc was significantly less effective than CA + CGA, CA + Arc, and CA + CGA + Arc on Days 1–6, and CGA + Arc on Days 2–6 (Figure 3C and Table S6).

Treatments also significantly changed the morphology and/or confluency of breast cancer cells. Combination treatments appeared to have stronger effects in this regard (Figures S4 and S5).

### 3.4. Single CA, CGA, and the Combination of CA + CGA Show No Harmful Effects While Arc Treated Groups, Alone or in Combination, Show Cytotoxic Effects in Normal Mammary Epithelial Cells

**Figure 4:**
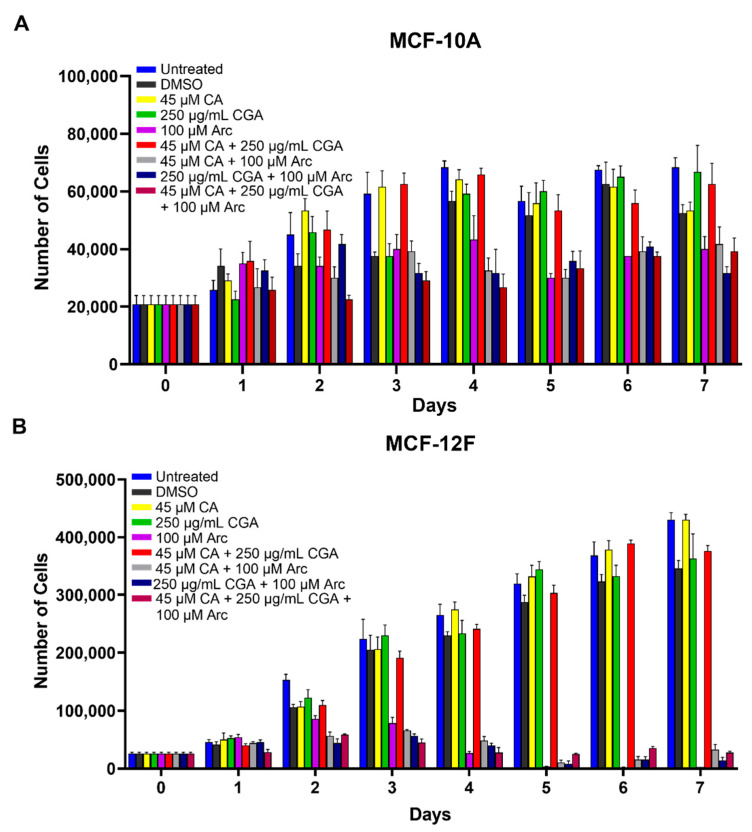
Normal mammary epithelial cell growth curves of (**A**) MCF-10A and (**B**) MCF-12F cells treated with 45 µM of CA, 250 µg/mL of CGA, and 100 µM of Arc, alone and in combination. Cell growth was determined by counting the number of cells in each group on each day for 7 days. Each bar represents the mean ± SEM of three biological replicates (*n* = 3). The groups were compared using a one-way ANOVA. The results of the statistical analysis are summarized in Tables S7 and S8. Cell growth differences between groups were considered significant at *p* ≤ 0.05.

To determine whether the chosen treatments had detrimental effects on normal cells, MCF-10A and MCF-12F normal mammary epithelial cells were treated with CA, CGA, and Arc, alone and in combination, for 7 days. Here, the highest IC_50_ concentrations across the three breast cancer cell lines were used. Altogether, CA, CGA, and the CA + CGA combination treatments showed no significant harmful effects on normal mammary epithelial cell growth. However, treatment groups containing Arc, alone and in combination, led to a significant reduction in both normal cell lines, with MCF-12F cells being more sensitive than MCF-10A cells. In MCF-10A cells, there were no significant differences in cell growth between the untreated group and the CA, CGA, and CA + CGA groups over the 7-day period. Arc and CA + Arc-treated MCF-10A cells had a significant cell growth reduction compared to the untreated group on Days 4–7, while the CGA + Arc and CA + CGA + Arc treatments significantly decreased the MCF-10A cell numbers on Days 3–7 (Figure 4A and Table S7).

In MCF-12F cells, the CA, CGA, and CA + CGA groups, again, performed very well in preserving normal cell growth, with the exception of Day 2, where CA and CA + CGA had a slight, but significant cell count number difference compared to the untreated group, although not compared to the DMSO group. On consecutive days, however, a significant difference between these three groups and the untreated group was not observed. In contrast, the treatment groups that contained Arc significantly decreased MCF-12F cell growth on Days 2–7 (Figure 4B and Table S8).

In MCF-10A normal cells, cell morphology and confluency did not change with the CA, CGA, and CA + CGA treatments. A slight change in morphology and decrease in confluency for the treatments containing Arc was observed. In MCF-12F normal cells, there was no change in morphology or cell confluency for CA, CGA, and CA + CGA treatments, while treatments containing Arc led to a decrease in cell confluency, as well as elongation, shriveling, and detachment of cells (Figures S4 and S5).

**Figure 5:**
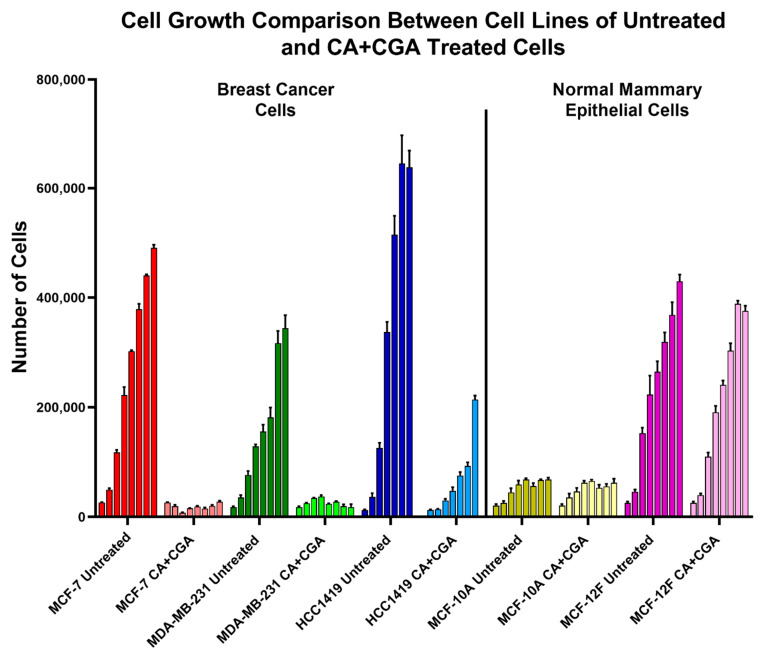
Cell growth comparison of untreated and CA + CGA-treated breast cancer and normal mammary epithelial cells. CA + CGA treatment significantly reduced breast cancer cell growth compared to untreated breast cancer cells, but did not cause cell growth reductions in normal mammary epithelial cells. Cell growth was determined by counting the number of cells in each group on each day for 6/7 days. Each bar represents the mean ± SEM of three biological replicates (*n* = 3).

Taking the breast cancer and normal mammary epithelial cell growth curves together, of all treatments tested, CA + CGA was shown to be the best candidate for breast cancer cell reduction as it was one of the strongest breast cancer cell growth inhibitors without causing normal cell cytotoxicity (Figure 5).

### 3.5. Effects of Single and Combination Treatments of CA, CGA, and Arc on Cell Death of Breast Cancer and Normal Mammary Epithelial Cells

Breast cancer and normal mammary epithelial cell death after treatment with CA, CGA, and Arc, individually and in combination, was explored by treating each cell line with the same concentrations from the previous cell growth experiments for 96 h. Cell death was evaluated twofold via flow cytometry: firstly by inspecting the presence of phosphatidylserine on the outer leaflet of the plasma membrane, a common indicator of apoptosis [98], by Annexin V-FITC staining, and secondly by staining cells with MitoTracker^TM^ Red CMXRos, which stains live cells based on their mitochondrial membrane potential (Δψm). Loss of fluorescence of MitoTracker^TM^ Red CMXRos is a sign of ΔΨm depolarization, which is involved in the apoptotic signaling pathway [99]. Loss of MitoTracker^TM^ Red CMXRos fluorescence is, therefore, another indicator of cell death.

**Figure 6:**
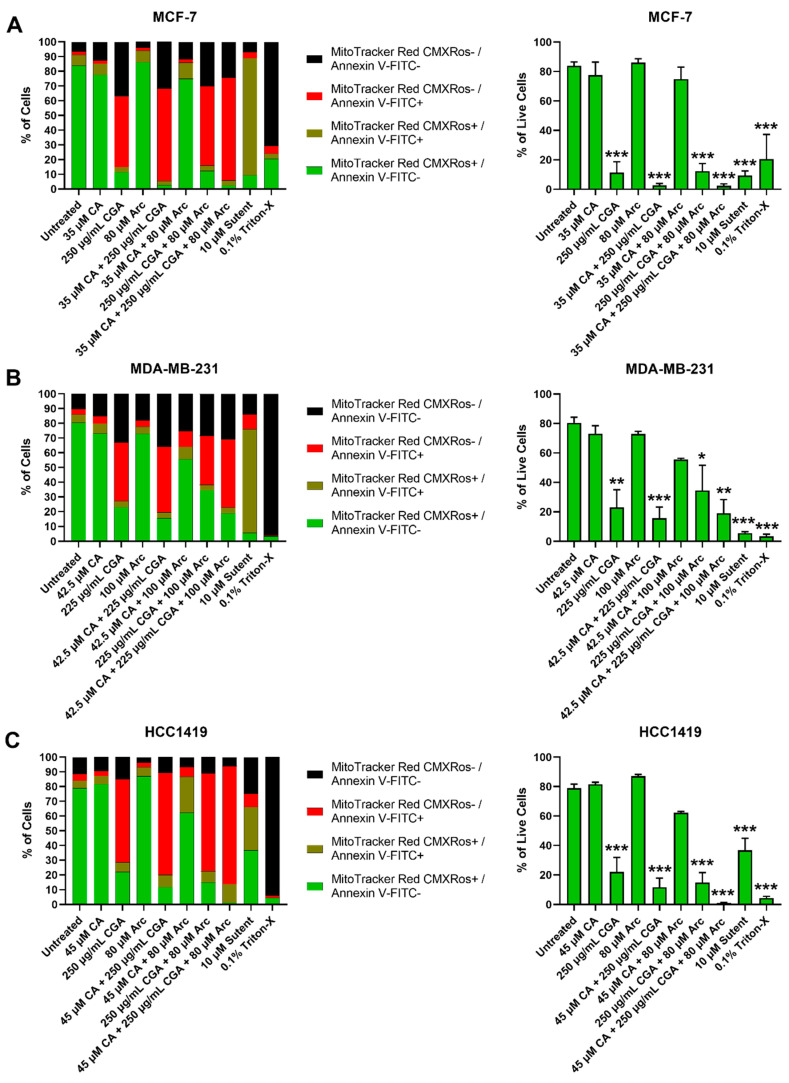
Cell death and live cell percentages in (**A**) MCF-7, (**B**) MDA-MB-231, and (**C**) HCC1419 breast cancer cells treated with CA, CGA, and Arc, alone or in combination, for 96 h. Green bars represent cells that are ΔΨm-positive and annexin V-negative (live cells); brown bars denote cells that are ΔΨm-positive and annexin V-positive (early cell death); red bars show cells that are ΔΨm-negative and annexin V-positive (late cell death); and black bars represent cells that are ΔΨm-negative and annexin V-negative (late cell death). Each green bar in the % live cell graphs represents the mean of means ± SEM from 3 biological and experimental replicates (*n* = 3) showing the percentage of live cells at 96 h. Twenty-four-hour 10 µM Sutent treatment was used as a positive control for Annexin V-FITC and 30 min 0.1% Triton-X treatment was used as a double-negative staining control. The groups were compared using a one-way ANOVA. Differences in the percentage of live cells between groups were considered significant at *p* ≤ 0.05. Statistical significance compared to untreated: * = *p* < 0.05; ** = *p* < 0.01; *** = *p* < 0.001.

In MCF-7 breast cancer cells, CA, Arc, and CA + Arc treatments did not cause significant cell death compared to the untreated group. However, CGA, CA + CGA, CGA + Arc, and CA + CGA + Arc-treated cells showed losses of ΔΨm, as well as a high percentage of cells with detectable phosphatidylserine. The CA + CGA and CA + CGA + Arc treatments caused the greatest reduction in live cells, with less than 5% of live cells remaining. However, there was no significant difference between these two groups and the other two CGA-containing groups CGA, and CGA + Arc (Figure 6A).

Similar results were seen in the treated MDA-MB-231 breast cancer cells, where the CA, Arc, and CA + Arc treatments did not lead to significant decreases in live cells compared to the untreated group. The treatment groups containing CGA showed a loss of ΔΨm and the presence of phosphatidylserine and led to significant cell death and reduction in live cells. The greatest live cell reduction was found in the CA + CGA treatment group, with only 15.6% of live cells surviving. We did not, however, note a significant difference between the CGA-containing treatment groups of CGA, CA + CGA, CGA + Arc, and CA + CGA + Arc (Figure 6B).

In the HCC1419 breast cancer cells, there was no significant difference in cell death between the CA, Arc, and CA + Arc-treated groups compared to the untreated groups. Like MCF-7 and MDA-MB-231, the treatment groups that contained CGA led to significant cell death in HCC1419 cells accompanied by the loss of ΔΨm and presence of phosphatidylserine. In this cell line, with only 1.1% live cells remaining, CA + CGA + Arc treatment caused the greatest reduction in live cells, but there was no significant difference between the CGA-containing groups (Figure 6C).

**Figure 7:**
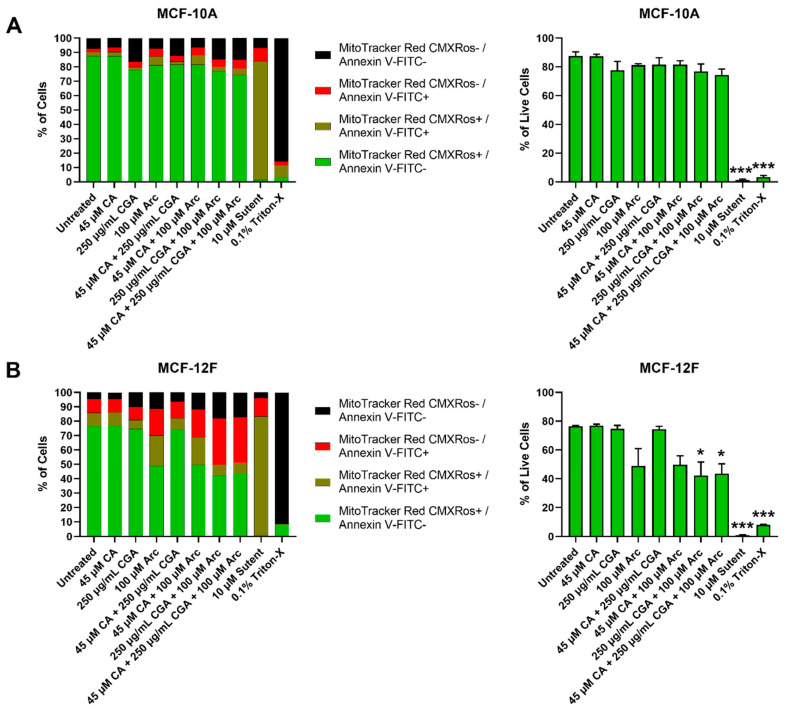
Cell death and live cell percentages in (**A**) MCF-10A and (**B**) MCF-12F normal mammary epithelial cells treated with CA, CGA, and Arc, alone or in combination, for 96 h. Green bars represent cells that are ΔΨm-positive and annexin V-negative (live cells); brown bars denote cells that are ΔΨm-positive and annexin V-positive (early cell death); red bars show cells that are ΔΨm-negative and annexin V-positive (late cell death); black bars represent cells that are ΔΨm-negative and annexin V-negative (late cell death). Each green bar in the % live cell graphs represents the mean of means ± SEM from 3 biological and experimental replicates (*n* = 3) showing the percentage of live cells at 96 h. Twenty-four-hour 10 µM Sutent treatment was used as a positive control for Annexin V-FITC and 30 min 0.1% Triton-X treatment was used as a double-negative staining control. The groups were compared using a one-way ANOVA. Differences in the percentage of live cells between groups were considered significant at *p* ≤ 0.05. Statistical significance compared to untreated: * = *p* < 0.05; *** = *p* < 0.001.

Strikingly, none of our treatments led to any significant cell death in the MCF-10A normal cells (Figure 7A). In MCF-12F normal cells, groups containing Arc led to increases in cell death; however, only the CGA + Arc and CA + CGA + Arc-treated cells caused statistically significant cell death compared to the untreated group. The lowest number of live cells was seen in these two groups, with only 42.3% and 43.5% of live cells remaining in the CGA + Arc and CA + CGA + Arc-treated cells, respectively. However, none of the Arc-containing treatment groups were statistically different from each other (Figure 7B). Surprisingly, single Arc treatment led to a higher percentage of cell death in the MCF-12F normal cells compared to any of the cancer cells.

Altogether, normal mammary epithelial cells showed lower cell death rates after 96 h of treatment compared to breast cancer cells, except for the Arc-containing groups in MCF-12F cells. Treatments that contain CGA were most effective in causing cell death in breast cancer cells. The CGA and CA + CGA treatment groups were most effective in inducing breast cancer cell death without significant harm to normal mammary epithelial cells. CA + CGA treatment showed, overall, lower live cell percentages in each breast cancer cell line compared to single CGA treatment (MCF-7 − CGA: 11.4% vs. CA + CGA: 2.7%; MDA-MB-231 − CGA: 23.0% vs. CA + CGA: 15.6%; HCC1419 − CGA: 22.1% vs. CA + CGA: 11.7%). These differences were, however, not statistically significant. Furthermore, in the MCF-10A normal cells, CA + CGA treatment actually showed a slightly higher percentage, although statistically insignificant, of live cells compared to CGA treatment (CGA: 77.7% vs. CA + CGA: 81.6%). In the MCF-12F normal cells, the percentage of live cells was nearly equal between CGA and CA + CGA-treated cells (CGA: 74.7% vs. CA + CGA: 74.4%). Therefore, CA + CGA treatment showed greater potency over single CGA treatment in facilitating breast cancer cell death while preserving normal mammary epithelial cells.

### 3.6. Effects of CA, CGA, and Arc, Alone and in Combination, on General Cell and Mitochondrial Morphology in Breast Cancer and Normal Mammary Epithelial Cells

The effects of the CA, CGA, Arc, CA + CGA, CA + Arc, CGA + Arc, and CA + CGA + Arc treatments on the general cell morphology, as well as the mitochondria and nucleus in MCF-7 breast cancer and MCF-10A normal cells, was analyzed by fluorescence, as well as phase–contrast and brightfield microscopy. The cells were stained with MitoTracker^®^ Green FM to visualize the mitochondria and Hoechst 33342 or DAPI to visualize the nucleus. Cell morphology was visualized using phase–contrast or brightfield microscopy. MitoTracker^®^ Green FM, unlike the MitoTracker^TM^ Red CMXRos used in the cell death assay, is independent of the mitochondrial membrane potential. It binds to free thiols on mitochondrial proteins and is used to depict mitochondrial mass [100].

**Figure 8:**
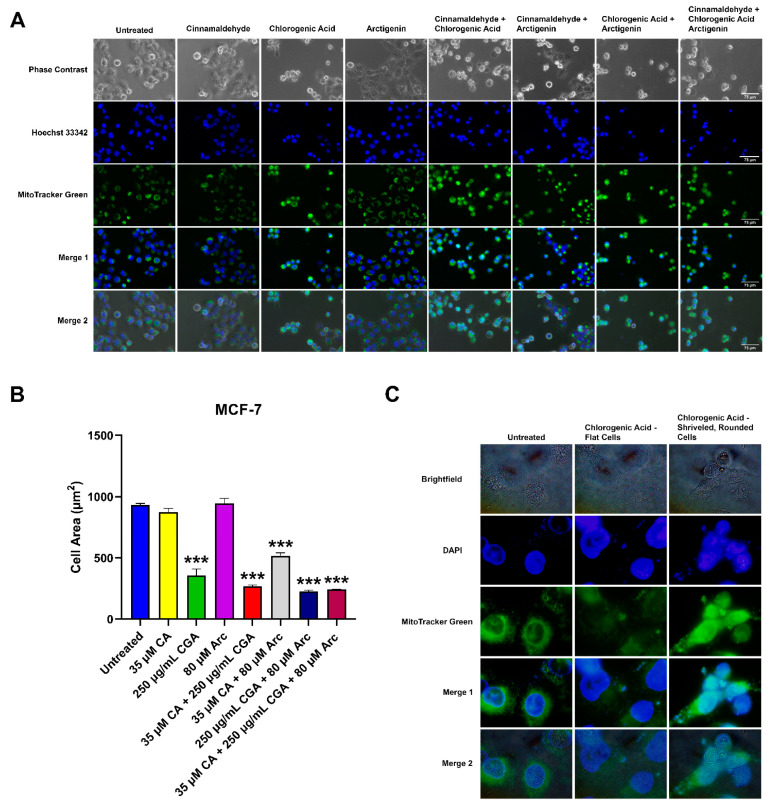
Cellular, mitochondria, and nuclear morphologies of MCF-7 breast cancer cells treated with 35 µM CA, 250 µg/mL CGA, and 80 µM Arc, alone and in combination, for 24 h. (**A**) Representative fluorescence microscopy images of MCF-7 breast cancer cells treated with CA, CGA, and Arc, alone and in combination, for 24 h. MCF-7 cells were stained with final concentrations of 150 µM MitoTracker^®^ Green FM and 1 μg/mL of Hoechst 33342 before visualization at 40× magnification (scale bar = 75 µm). (**B**) Cell area measured from 40× phase–contrast images of MCF-7 cells treated with CA, CGA, and Arc, alone and in combination, for 24 h. Bars represent the cell area mean values ± SEM of three experimental replicates (*n* = 3). Statistical significance compared to untreated: *** = *p* < 0.001. (**C**) Representative fluorescence microscopy images of MCF-7 cells treated with 250 µg/mL CGA for 24 h at 100× magnification. MCF-7 cells were stained with a final concentration of 150 µM MitoTracker^®^ Green FM and were mounted with DAPI/Antifade (0.4 μg/mL of DAPI) before visualization at 100× magnification.

After 24 h of treatment, the most significant changes in the cellular, mitochondrial, and nuclear morphologies were noted in the CGA, CA + CGA, CGA + Arc, and CA + CGA + Arc treated groups. The cells in these groups underwent significant decreases in cell size characterized by cell shrinkage, rounding, and bulging up, as well as membrane blebbing. The mitochondria in these groups appeared diffused as well as clustered, clumped, and aggregated. Unlike in the CGA-containing combination groups, the single CGA-treated group showed two morphologically distinct cell populations. One set of cell populations showed cell morphologies similar to CA + CGA, CA + Arc, and CA + CGA + Arc-treated cells. However, the second set of cell populations had flattened cells with mitochondria that were almost completely diffused and almost undetectable signal intensity. It is possible that either these two cell populations undergo different cell death mechanisms, or one cell population predates the other in the time of death, with the shriveled, rounded cells having died earlier than flat cells from the time of imaging. The CA + Arc treated MCF-7 cells also showed two cell populations, with some rounded and some flat. However, rounded cells did not appear as shriveled as those in the treatment groups containing CGA. Furthermore, mitochondria did not appear diffused, but rather clustered to one side of the cells. The CA and Arc single treatments did not show any significant changes in cellular, mitochondrial, or nuclear morphology (Figure 8A–C).

The six-hour treatments of the individual and combination treatments in MCF-7 cells did not lead to any significant changes in the cellular, mitochondrial, or nuclear morphologies, except for CA + CGA + Arc. The phase–contrast images of CA + CGA + Arc treatment showed several cells that were shrunk and rounded up, as well as blebbing membranes (Figure S6A).

**Figure 9:**
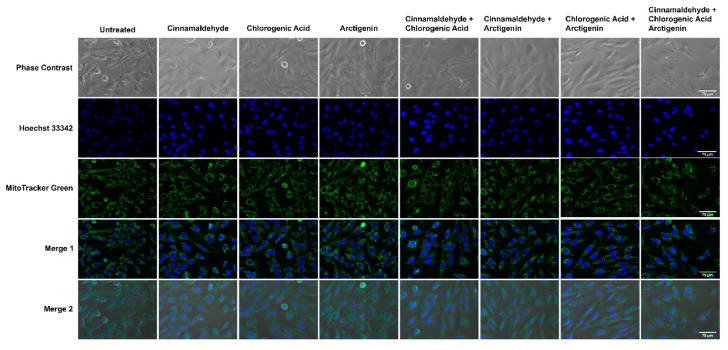
Representative fluorescence microscopy images of MCF-10A cells treated with 45 µM CA, 250 µg/mL CGA, and 100 µM Arc, alone and in combination, for 24 h. After 24 h treatment, cells were stained with final concentrations of 150 µM MitoTracker^®^ Green FM and 1 μg/mL of Hoechst 33342 before visualization at 40× magnification (scale bar = 75 µm).

In contrast to the treated MCF-7 breast cancer cells, none of the treatments in the MCF-10A normal mammary epithelial cells led to significant changes in the cellular, mitochondrial, or nuclear morphologies at 6 h (Figure S6B) or 24 h (Figure 9). However, we noticed an increase in the dark spots on top of the cell bodies in the Arc-containing treatment groups that appeared more prominent at 24 h compared to the 6 h treatments. These spots did not appear to be co-localized with the mitochondria. At this time, the role of these dark spots could not be identified. One possibility is an increase in the number of vacuoles in MCF-10A cells treated with Arc-containing groups. Vacuolization often occurs in response to changing environments, which can lead to cell death [101,102]. In this study, we previously showed a reduction in MCF-10A cell growth in the Arc-containing treatment groups over a period of 7 days, in which vacuolization may be an early occurrence post-treatment.

### 3.7. Effects of Single and Combination Treatments of CA, CGA, and Arc on Mitochondrial Superoxide Production in Breast Cancer and Normal Mammary Epithelial Cells

Additionally, the effects of single CA, CGA, and Arc treatments, as well as their combinations, on the production of mitochondrial superoxide in MCF-7 breast cancer cells and MCF-10A normal mammary epithelial cells were examined. The control and treated cells were stained with MitoSOX^TM^ Red, which fluoresces red in the presence of mitochondrial superoxide. Superoxide (O_2_^•−^) is one of several known reactive oxygen species (ROS) occurring in biological systems. This anion is generated by a one-electron reduction of dioxygen (O_2_). The oxidative stress in cells caused by superoxide and other ROS are activators of apoptosis and other cell death mechanisms, including autophagy [103,104,105]. Indeed, raising ROS in cancer cells is one of many strategies employed by anti-cancer drugs to induce cancer cell death [103,106,107,108,109]. However, many of them also cause oxidative stress in normal cells [110,111,112].

**Figure 10:**
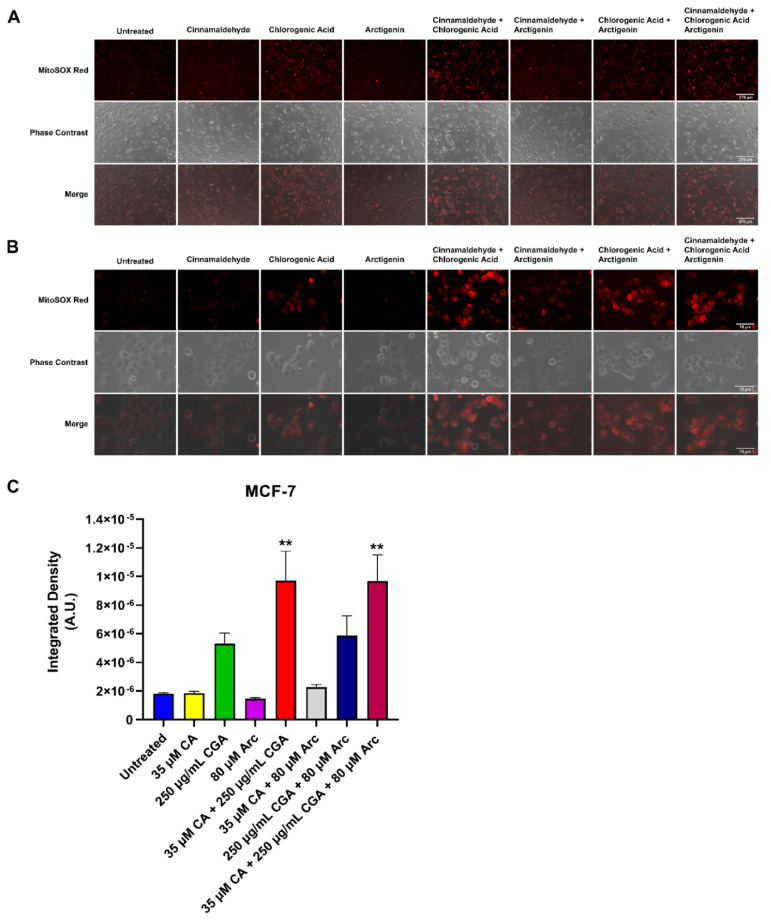
Superoxide production in MCF-7 breast cancer cells treated with 35 µM CA, 250 µg/mL CGA, and 80 µM Arc, alone and in combination, for 2 h. (**A**) Representative fluorescence microscopy images of MCF-7 cells treated with CA, CGA, and Arc, alone and in combination, for 2 h at 10× magnification (scale bar = 275 µm). (**B**) Representative fluorescence microscopy images of MCF-7 cells treated with CA, CGA, and Arc, alone and in combination, for 2 h at 40× magnification (scale bar = 75 µm). After 2 h of treatment, cells were stained with 5 μM MitoSOX™ Red before visualization. (**C**) Bars represent integrated mean density values ± SEM in arbitrary units (A.U.) of three experimental replicates (*n* = 3) based on MitoSOX™ Red fluorescence. Groups were compared using a one-way ANOVA. Differences in superoxide production between groups were considered significant at *p* ≤ 0.05. Statistical significance compared to untreated: ** = *p* < 0.01.

Here, superoxide generation was evaluated using fluorescence microscopy, and the integrated density was calculated to quantify the presence of superoxide. MCF-7 and MCF-10A cells were treated for 2 h with respective treatment concentrations as previously used. In MCF-7 breast cancer cells, the groups containing CGA increased superoxide in comparison to the untreated group. However, only the CA + CGA and CA + CGA + Arc-treated groups showed a statistically significant increase compared to the untreated group across three experimental replicates. CGA and CGA + Arc were neither statistically different from the untreated group, nor from CA + CGA and CA + CGA + Arc. CA and Arc treatments did not lead to any rise in superoxide production in MCF-7 breast cancer cells, and CA + Arc treatment only showed a slight, but non-significant, elevation (Figure 10A–C).

**Figure 11:**
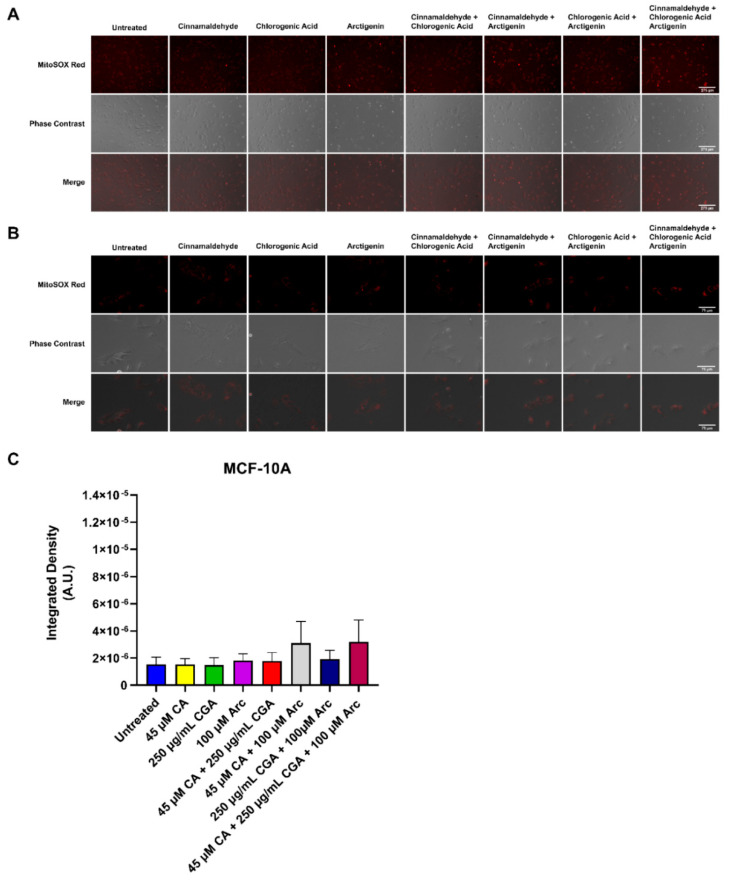
Superoxide production in MCF-10A normal mammary epithelial cells treated with 45 µM CA, 250 µg/mL CGA, and 100 µM Arc, alone and in combination, for 2 h. (**A**) Representative fluorescence microscopy images of MCF-10A cells treated with CA, CGA, and Arc, alone and in combination for 2 h at 10× magnification (scale bar = 275 µm). (**B**) Representative fluorescence microscopy images of MCF-10A cells treated with CA, CGA, and Arc, alone and in combination, for 2 h at 40× magnification (scale bar = 75 µm). After 2 h treatment, cells were stained with 5 μM MitoSOX™ Red before visualization. (**C**) Bars represent integrated density mean values ± SEM in arbitrary units (A.U.) of three experimental replicates (*n* = 3) based on MitoSOX™ Red fluorescence. Groups were compared using a one-way ANOVA. Differences in superoxide production between groups were considered significant at *p* ≤ 0.05.

In the MCF-10A mammary epithelial cells, there was no statistically significant difference in superoxide production between any of the treatments and the untreated group across three experimental replicates. Additionally, no significant difference was observed between treatments. CA + Arc and CA + CGA + Arc treatments led to an increase in the superoxide levels in one of the three repeated experiments, but this was not statistically different across all three experimental replicates (Figure 11A–C).

Overall, CA + CGA was the most potent treatment in raising breast cancer cell superoxide and did not significantly increase normal cell superoxide formation. CA + CGA + Arc was equally as potent as CA + CGA in increasing superoxide in breast cancer cells. However, we observed a slight, although not statistically significant, increase in superoxide for this treatment in normal cells, most likely driven by combining CA with Arc, as the CA + Arc treatment group had almost identical superoxide levels compared to CA + CGA + Arc in normal cells.

### 3.8. Effects of CA, CGA, Arc, CA + CGA, and CA + CGA + Arc Treatments on Cellular ATP Production by Glycolysis and Oxidative Phosphorylation in Breast Cancer Cells

Here, a bioenergetic profile of CA, CGA, Arc, CA + CGA, and CA + CGA + Arc-treated MCF-7 breast cancer cells after 48 h treatment was established. The Agilent^TM^ Seahorse XF Real-Time ATP Rate Assay was utilized to measure the amount of ATP produced by glycolysis as well as oxidative phosphorylation. Due to the limited well space on the assay plate, only the most relevant treatment groups from previous experiments were employed. This assay relies on the measurements of the oxygen consumption rate (OCR) and extracellular acidification rate (ECAR) at baseline (0–18.7 min), after oligomycin injection (27.5–44.7 min), and after rotenone + antimycin A injection (53.4–70.5 min). This allows for the quantification of the total, mitochondrial, and glycolytic ATP production rates. Oligomycin inhibits ATP synthase, resulting in a decrease in OCR that is equivalent to the ATP produced by the mitochondria. ECAR measurements quantify glycolytic ATP production based on the acidification of the assay medium due to the release of one H^+^ per lactate during glycolysis. Because oxidative phosphorylation also causes assay medium acidification, rotenone + antimycin A is injected to shut down mitochondrial respiration. ECAR measurements following rotenone + antimycin A injection are subtracted to allow for the calculation of ECAR from glycolysis alone. Glycolytic ECAR and the buffer factor of the assay medium then allowed for the calculation of the proton efflux rate (PER), which quantifies the glycolytic ATP production rate. Figure S7A–C shows the OCR, ECAR, and PER measurements at each time point for each treatment group after 48 h of treatment.

**Figure 12:**
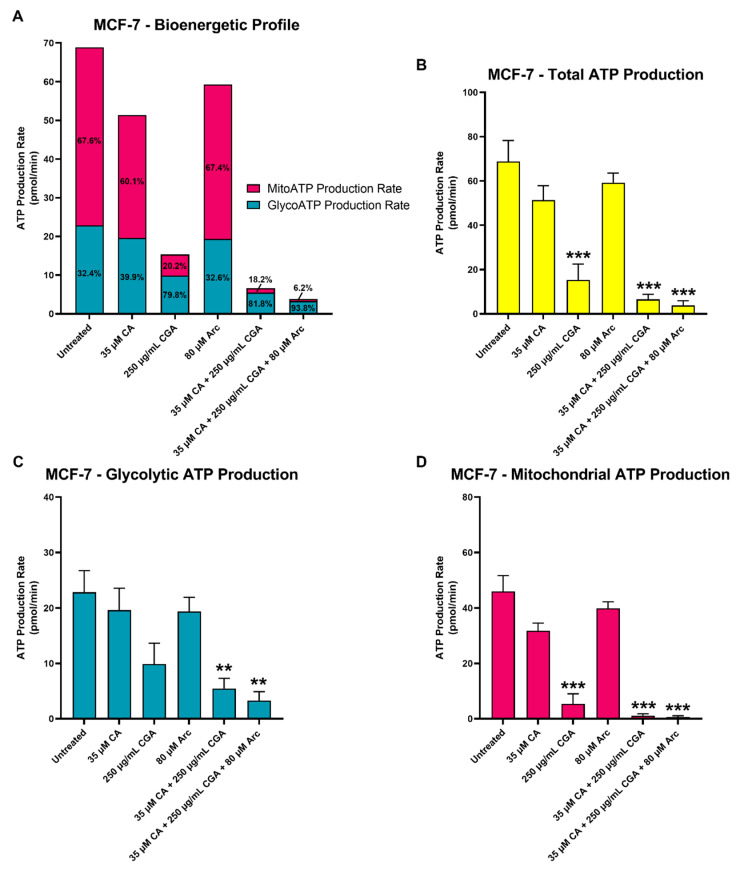
Bioenergetic profile and total, glycolytic, and mitochondrial ATP production in MCF-7 breast cancer cells treated with 35 µM CA, 250 µg/mL CGA, and 80 µM Arc, for 48 h. (**A**) Each bar represents the mean of mean ATP production rate (pmol/min) values from 3 biological and 4 experimental replicates (*n* = 4). Percentages denote the proportion of the total ATP production rate generated by the mitochondria (red) and by glycolysis (blue). (**B**) Total, (**C**) glycolytic, and (**D**) mitochondrial ATP production rates in MCF-7 breast cancer cells. Each bar represents the mean ± SEM of the ATP production rate (pmol/min) values from 3 biological and 4 experimental replicates (*n* = 4). Groups were compared using a one-way ANOVA. Differences in the total, glycolytic, and mitochondrial ATP production between groups were considered significant with *p* ≤ 0.05. Statistical significance compared to untreated: ** = *p* < 0.01; *** = *p* < 0.001.

CGA, CA + CGA, and CA + CGA + Arc significantly reduced the total ATP production rates of MCF-7 breast cancer cells to 15.3, 6.6, and 3.9 pmol/min, respectively, compared to 68.8 pmol/min in the untreated group. There was no statistical significance between these three treatment groups. With 51.3 and 59.3 pmol/min, respectively, CA and Arc also showed decreased total ATP production rates. However, this reduction was not statistically significant from that of the untreated group (Figure 12A). In the untreated group, 32.4% of the total ATP came from the mitochondria, while 67.6% was generated by glycolysis. CA and Arc treatments caused similar mitoATP:glycoATP ratios compared to the untreated group, where CA treatment showed 60.1% mitoATP and 39.9% glycoATP, and Arc showed 67.4% mitoATP and 32.6% glycoATP. Therefore, in the untreated, and CA and Arc-treated groups, the majority of the ATP of MCF-7 breast cancer cells was generated by oxidative phosphorylation. Interestingly, in treatments containing CGA, this ratio completely flipped, where glycoATP accounts for the majority of the total ATP produced in the MCF-7 cells. In the CGA-treated cells, the ratio of mitoATP:glycoATP was 20.2%:79.8%; in the CA + CGA-treated cells, 18.2%:81.8%; and CA + CGA + Arc-treated cells, 6.2%:93.8% (Figure 12A). This suggests that the CGA, CA + CGA, and CA + CGA + Arc treatments more strongly inhibit the mitochondrial ATP production rates compared to the ATP production rates from glycolysis in MCF-7 cells. This is also evidenced in Figure 12B,C showing the respective glycoATP and mitoATP production rates upon treatment. The CGA, CA + CGA, and CA + CGA + Arc treatments decreased the ATP production rates in greater magnitudes in mitoATP than in glycoATP compared to the untreated group. However, even the glycoATP production rates were significantly reduced in the CA + CGA, and CA + CGA + Arc-treated MCF-7 cells compared to the untreated group, whereas CGA only significantly reduced mitoATP production. Arc treatment showed only slight decreases in the mitoATP and glycoATP production rates. However, these reductions were not statistically significant. In CA-treated cells, the mitoATP and glycoATP production rates also decreased, but were not statistically significant. However, the mitoATP production rate was reduced at higher magnitudes compared to glycoATP (Figure 12B,C).

Altogether, CA + CGA and CA + CGA + Arc were most effective in significantly reducing the total, mitochondrial, and glycolytic ATP production rates in MCF-7 breast cancer cells. Interestingly, mitochondrial ATP production was more strongly impaired by the treatment groups, with the exception of Arc, than glycolytic ATP production.

## 4. Discussion

One of the many challenges of current chemotherapeutics that are used to treat breast cancer is their cytotoxic effects on normal cells, leading to side effects in cancer patients. This is because chemotherapy drugs often target cell division [113], which affects normal cells as well as cancer cells, leading to toxic effects that are systemic. These effects include anemia, constipation, diarrhea, difficulty swallowing, fatigue, fertility problems, hand–foot syndrome, hair loss, increased risk of infection, loss of appetite, nausea and vomiting, nerve and muscle problems, permanent heart damage, skin changes, and urine and bladder changes [114,115]. Another drawback of chemotherapeutics is the ability of cancer cells to develop chemoresistance, thus evading cell death. Considering these challenges, the aim of this work was to explore alternative breast cancer treatments that are both effective and non-cytotoxic to normal cells.

Herein, we investigated three plant-derived chemicals, namely cinnamaldehyde (CA), chlorogenic acid (CGA), and arctigenin (Arc), alone and in combination, as possible anti-breast cancer agents that show minimal cytotoxicity to normal cells. Here, we report that the combination of cinnamaldehyde with chlorogenic acid (CA + CGA) was the most promising treatment in promoting breast cancer cell death without causing significant harmful effects on normal cells. CA + CGA effectively reduced breast cancer cell growth, mitochondrial integrity, and ATP production, as well as induced cell death and superoxide production. At the same time, it did not decrease normal mammary epithelial cell growth, disturb mitochondrial integrity, promote cell death, or increase the superoxide levels in these cells. CA + CGA was a more effective anti-breast cancer agent than any of the three phytochemicals alone as well as the other two double combinations (CA + Arc and CGA + Arc). CA + Arc and CGA + Arc, however, led to significant cytotoxic effects in normal cells. The triple combination (CA + CGA + Arc) was shown to be as potent or greater in inducing breast cancer cell death compared to CA + CGA. However, the higher potency was not statistically significant from that of CA + CGA treatment, except in the HER2-overexpressing breast cancer cell line HCC1419. Similar to CA + Arc and CGA + Arc, this triple combination was found to exert significant cytotoxic effects on normal cells. Therefore, patients with breast cancers that overexpress HER2, which constitute about 10–30% of all breast cancers [116], could perhaps benefit from this treatment in the future; however, its more harmful effects on normal mammary epithelial cells need to be considered.

Overall, our findings supported our hypothesis that combination treatments elicit higher anti-breast-cancer responses than using single components alone. Several other studies have investigated plant- and/or dietary-derived compounds in combination, including those used in our study, as potential anticancer agents and have reached similar conclusions [20,52,82,84,117,118], attesting to the potential of this strategy to combat cancer. The benefits of combination treatments of phytochemicals may also stem from the ability to increase the number of pathways that can be targeted at once, which may reduce the chemoresistance of cancer cells [18].

It is of note, however, that CGA treatment alone was an effective inducer of cell death and mitochondrial disturbance in breast cancer cells and did not promote cytotoxic effects in normal mammary epithelial cells. This supports previous work by Deka et al. [39], which showed the involvement of the mitochondria in breast cancer cell death, and Zeng et al. [43], who found no adverse effects of CGA in MCF-10A normal mammary epithelial cells. CA and Arc were found to reduce breast cancer cell growth, but, surprisingly, did not induce cell death that is dependent on the mitochondrial membrane potential or is associated with increases in superoxide levels. This is contrary to what was hypothesized based on previous studies. CA treatment was found to cause apoptosis in breast cancer cells [36] as well as in other cancer cells through mitochondrial-dependent pathways, and increases in ROS [56,58,71]. In addition, CA *Cinnamomum cassia* and *Cinnamomum zeylanicum* extracts, of which CA is a major component, were shown to decrease the mitochondrial membrane potential and elevate ROS in breast cancer cells [35,37]. Similarly, a previous study also demonstrated the induction of apoptosis by Arc in breast cancer cells [44]. Moreover, studies on the effects of Arc treatment on several other cancers have demonstrated that Arc treatment leads to cell death, mitochondrial membrane potential changes, increases in ROS, and decreases in ATP production [63,64,65,66,67,68,69]. Interestingly, these findings were primarily demonstrated in cancer cells that were either glucose-starved, acidity-tolerant, or oxidative phosphorylation-dependent (rather than glycolytic). This may, in part, explain our findings that Arc did not cause mitochondrial-dependent cell death in our breast cancer cells. It is of note, however, that, in our study, MCF-7 cells were found to generate more ATP from oxidative phosphorylation than glycolysis. It is possible that CA and Arc decrease breast cancer cell proliferation without causing cell death, or that a different, mitochondrial-independent cell death mechanism is responsible for the decrease in breast cancer cell growth. Further research is needed to investigate this. CA was not observed to cause adverse effects on the normal mammary epithelial cells MCF-10A and MCF-12F at a concentration of 45 µM. This is similar to findings by Wani et al. that did not find a significant decrease in MCF-10A viability at 40 µM. However, their study noted a reduction in MCF-10A cells at increasing concentrations [34].

One of the more surprising findings of our study was Arc-induced cytotoxicity in normal mammary epithelial cells. Arc treatments, alone and in combination, promoted significant adverse effects in MCF-10A and MCF-12F normal mammary epithelial cells, with MCF-12F cells being more susceptible to them. Based on the literature, most studies that have examined the effects of Arc on normal cells in conjunction with investigating its effects on cancer cells found no adverse effects of Arc on normal cells. The normal cells used in these studies included normal hepatic LO2 cells, normal colon CCD-18Co cells, normal prostate epithelial PrEC, RWPE-1, and HPrEC cells, normal lung fibroblast IMR-90 and WI-38 cells, fibroblast KMST-6 cells, and embryo fibroblast OUMS-36T-5F cells [63,68,91,92,93]. In addition, Arc did not reduce H184B5F5/M10 normal mammary epithelial cell proliferation while reducing MDA-MB-231, but not MCF-7, cell proliferation at the same concentrations. Similarly, another study found that MCF-10A was less sensitive to Arc treatment compared to MDA-MB-231, MDA-MB-468, MDA-MB-435S, MDA-MB-453, and MDA-MB-231, but more susceptible than MCF-7 and SK-BR3 breast cancer cells [31,44]. We observed that MCF-10A was about as equally susceptible to Arc as MCF-7, MDA-MB-231, and HCC1419 cells regarding the degree of cell death. MCF-12F cells, on the other hand, were more sensitive than breast cancer cells. More research is needed to investigate this occurrence and why normal mammary epithelial cells may be more sensitive to Arc compared to the other normal cell lines studied. Perhaps breast cancer cells are more resistant to Arc than other cancers; exceedingly high concentrations are needed to kill breast cancer cells, making it also cytotoxic to normal mammary epithelial cells. This could indicate that Arc may not be advantageous in selectively killing cancers of the breast. The effects of Arc could, however, be explored in connection with CA and CGA in other cancer and normal cells of different tissues, where results may be more promising.

Breast cancer cells treated with single CGA or combinations containing CGA were found to undergo significant rates of cell death. More research is needed to confirm the exact cell death mechanism of these treatments, especially CGA as well as CA + CGA. In this study, breast cancer cells treated with CGA alone or in combination exhibited a loss in ΔΨm, and the presence of phosphatidylserine was confirmed. While the presence of phosphatidylserine measured by annexin V during flow cytometry may suggest apoptosis as the main cell death mechanism; necrosis, necroptosis, and other cell death mechanisms are also a possibility. This is because phosphatidylserine is not only present on the outer leaflet of the cell membrane during apoptosis, but can also be detected by annexin V when the cell membrane is impaired. While disruptions in mitochondria and the mitochondrial membrane potential, as well as the production of ROS, are known to occur with apoptosis, these characteristics may also take place during necroptosis, autophagy, and other cell death mechanisms [119,120,121]. As stated earlier, the CGA-only-treated cells were found to have two phenotypically distinct cell populations at 24 h, while the combination CGA treatments largely caused one cell death population similar to one of the two populations in CGA-only-treated MCF-7 cells. This population, based on phase–contrast and fluorescence microscopy imaging, demonstrated cell shrinkage and membrane blebbing, which are characteristic of apoptosis [122,123], whereas necrosis and necroptosis usually cause cell swelling. Paraptosis does not usually involve membrane blebbing [124], while autophagy does not usually cause cell shrinkage [122]. Regarding the second morphologically distinct cell population in the CGA-only-treated MCF-7 cells, phase–contrast and fluorescence microscopy images indicated no cell shrinkage or membrane blebbing, but a reduction in mitochondrial signal. Either these cells are at an earlier stage of cell death and will undergo the same cell death mechanism as the second cell population, or were experiencing a different cell death mechanism, such as autophagy, that causes mitochondrial degradation [122]. It is of note that a previous study identified apoptosis as a cell death mechanism of CGA-induced breast cancer cell death [39]. Further investigations are needed to determine the exact cell death mechanism in breast cancer cells treated with CGA, CA + CGA, CGA + Arc, and CA + CGA + Arc.

The ATP production rate assay revealed that MCF-7 breast cancer cells generally derived more ATP from oxidative phosphorylation compared to glycolysis, which is in line with the observations of Romero et al. [125]. CA + CGA, as well as CA + CGA + Arc treatments, were found to significantly decrease the total, mitochondrial, and glycolytic ATP production rates in MCF-7 breast cancer cells, while the CGA-only treatment significantly decreased the total and mitochondrial ATP. These findings suggest an advantage of using combinatorial CGA, rather than CGA alone to target the metabolism of breast cancer cells. Previous studies have shown that cancer cells that derive their energy predominantly from oxidative phosphorylation are able to resist treatments that target it by switching to glycolysis and vice versa [4,15,16]. Therefore, using combinatorial CGA might address the current challenges of targeting the metabolism of cancer cells due to metabolic flexibilities that cancer cells often exhibit, leading to treatment resistance.

Strikingly, CA + CGA treatment was able to induce superoxide production in breast cancer cells without doing so in normal mammary epithelial cells. In the early stages of tumorigenesis, increased ROS aid in malignant transformation [7,126,127]. However, eventually, cancer cells are forced to elevate their antioxidant systems in order to maintain survival under elevated ROS levels [128,129,130,131], which are generally higher compared to those in normal cells [132]. This trademark of cancer cells leaves them susceptible to anticancer treatments that increase ROS levels above their survival threshold [103,133,134]. Indeed, increased ROS levels induce apoptosis and autophagy in cancer cells [103,104,105] and many chemotherapeutics elevate ROS levels to induce cancer cell death [103,107,108,109]. However, many of these drugs also raise the ROS levels in normal cells, leading to the cytotoxic effects seen with these drugs [110,111,112]. Therefore, selectively targeting and increasing ROS production in cancer cells, but not in normal cells, has been regarded as a promising strategy to combat cancer [103,106]. There have been some concerns in using antioxidants as potential anticancer agents due to cancer cells employing antioxidant systems to negate their increased levels of ROS [103,135,136,137]. Therefore, some antioxidants may actually promote further tumor progression. However, the possible dangers of using antioxidants as anticancer agents appear to primarily pertain to those that decrease the ROS levels in cancer cells. Antioxidants that stabilize ROS levels in normal cells and raise them in cancer cells should be considered a desirable feature of anticancer agents due to their beneficial effects in normal cells. CA, CGA, and Arc, have all shown antioxidant capacities in normal cells in previous studies [21,22,23,24,25,26,27,28,29,30], but their antioxidant effects appear to be limited to normal cells and were not shown in cancer cells.

On the other hand, CA, CGA, and Arc have previously been shown to induce ROS in cancer cells, leading to cell death [44,59,60,64,70,71,138,139]. In contrast to previous studies, our study found that CA and Arc, as well as the combination of both, did not significantly increase the superoxide levels in breast cancer cells. Moreover, we observed a slight, but not statistically significant, increase in superoxide in normal cells treated with CA + Arc. It is possible, however, that CA and/or Arc increase other reactive oxygen species or reactive nitrogen species that were not analyzed in the breast cancer cells to induce cell death. We did, however, note an uptick in the superoxide levels in CGA-treated breast cancer cells. However, this finding was not statistically significant. CGA did not lead to any increases in the superoxide levels in normal cells. CA + CGA and CA + CGA + Arc were the only two treatments found to significantly increase the superoxide levels in almost identical measures in breast cancer cells. CA + CGA + Arc was observed to slightly, but not significantly, raise the superoxide levels in normal cells. Of all the treatments tested, CA + CGA was the most promising in selectively increasing the ROS levels in breast cancer cells, but not in normal mammary epithelial cells. These findings suggest the promising potential of CA + CGA as a selective ROS inducer in breast cancer cells without raising the ROS levels in normal cells. For this reason, CA + CGA may have an advantage over traditional chemotherapeutic drugs by selectively killing breast cancer cells by increasing their ROS levels without doing so in normal cells, thereby potentially reducing the side effects in breast cancer patients.

Two signaling pathways that could be explored in future studies with regard to the synergistic effects of CA + CGA and its selective superoxide/ROS increase in breast cancer cells, but not normal mammary epithelial cells, are nuclear factor–erythroid factor 2-related factor 2 (Nrf2) and Hypoxia-inducible factor 1 (HIF-1) signaling. Nrf2 is a redox-sensitive and antioxidant master transcription factor. It assists in maintaining and restoring redox homeostasis in cells, and is able to negate increases in mitochondrial ROS production [140]. Cancer cells have been shown to maintain this pathway in order to ensure survival under higher levels of ROS compared to normal cells [103,131]. CA and CGA by themselves have been associated with Nrf2 signaling in previous studies. CA has been shown to induce Nrf2 signaling in several cancer cells, including breast cancer, colon cancer, and liver cancer cells [141,142,143,144]. Studies examining the effects of CGA on Nrf2 signaling have been limited to studies in normal cells, in which CGA was shown to exhibit cytoprotective effects by reducing oxidative stress and restoring redox homeostasis through the involvement of Nrf2 [23,24,27,28]. Future studies may benefit from investigating the effects of CGA, alone, on Nrf2 activity in cancer cells. Since our study did not find a significant increase in superoxide production in CA-treated breast cancer cells, this might explain why CA treatment on its own leads to an increase in Nrf2 activity in breast cancer. CA treatment on its own might not be potent enough to render breast cancer cells unable to negate accumulating ROS levels, allowing breast cancer cells to utilize antioxidant mechanisms through Nrf2 and thus avoiding cell death. CA + CGA, on the other hand, was found to increase the superoxide levels in breast cancer cells, without doing so in normal mammary epithelial cells. It is, therefore, possible that combining CA with CGA synergistically inhibits Nrf2 in breast cancer cells, instead of activating it, perhaps rendering breast cancer cells incapable of counteracting increasing ROS levels caused by CA + CGA treatment. Nrf2 inhibition by CA + CGA may, therefore, be a possible reason for the increased anti-breast-cancer activity of CA + CGA compared to those of CA or CGA alone. However, the possible Nrf2 inhibition by CA + CGA is speculative and needs to be tested in future studies. HIF-1 signaling is another possible target of CA + CGA and is overexpressed in many solid tumor cells under hypoxic conditions. HIF-1 is a key transcriptional regulator of cellular responses to low oxygen levels [145], and several studies have reported that CA and CGA by themselves inhibit HIF-1 signaling [146,147,148,149,150]. Even though HIF-1 is most commonly associated with cancer angiogenesis, it may also play an important role in cancer metabolism. Under hypoxic conditions, HIF-1 aids cancer cells in nullifying low oxygen levels and elevated ROS levels by acting on mitochondrial metabolism [151,152]. Furthermore, HIF-1 signaling has been shown to be involved in helping cells balance oxidative phosphorylation and glycolysis [152]. Therefore, the synergistic cytotoxic effects of CA + CGA in breast cancer cells may involve the dysregulation of HIF-1 signaling, leaving breast cancer cells defenseless to the harmful effects of hypoxia, increased ROS levels, and metabolic disturbances caused by CA + CGA treatment.

Further research into CA, CGA, and Arc combinations, especially CA + CGA, as adjuvants to chemotherapy should also be explored. Each compound has individually been shown to have synergistic anticancer effects with certain chemotherapeutic drugs [31,53,73,74,75,76,77,78,79,80,81]. Due to its selective killing of breast cancer cells, targeting their metabolism and increasing ROS, CA + CGA may enhance the efficacy of chemotherapeutics in breast cancer and decrease chemoresistance. At the same time, our study showed that CA + CGA did not increase the ROS levels in normal cells and may, therefore, be able to reduce the negative side effects by counterbalancing chemotherapeutic-enhanced ROS in normal cells, especially since CA and CGA have individually been shown to have antioxidant capacities in normal cells. However, the antioxidant capacities of CA + CGA in normal cells need to be evaluated in future studies. 5-FU, oxaliplatin, and lapatinib were among the chemotherapeutics potentiated by either CA or CGA [73,74,76,77]; therefore, testing the effects of CA + CGA combined with these chemotherapeutics in cancer and normal cells may show promising results.

## 5. Conclusions

This work highlights the potential of combining plant- and dietary-derived antioxidants in cancer therapy. Moreover, selectively disrupting the metabolism and ROS of cancer cells may be a promising strategy to kill cancer cells without cytotoxic effects on normal cells. This study has provided evidence that combining cinnamaldehyde with chlorogenic acid has promising anti-breast cancer potential. This treatment induced breast cancer cell death, accompanied by significant perturbations in mitochondrial integrity, increases in superoxide, and reductions in mitochondrial and glycolytic ATP production without significant cytotoxic effects in normal mammary epithelial cells. Thus, this combination treatment is an attractive anti-breast cancer candidate with the potential to improve current treatment strategies that may enhance the quality of life of cancer patients by reducing debilitating side effects. To our knowledge, this is the first study to combine cinnamaldehyde with chlorogenic acid as an effective anticancer agent without cytotoxicity to normal cells.

## Figures and Tables

**Table 1:** Flow cytometry channel voltages in each cell line.

Cell Lines	FSC Voltage	SSC Voltage	BL1 Voltage	YL1 Voltage
MCF-7	150	300	185	290
MDA-MB-231	150	300	190	270
HCC1419	160	290	210	285
MCF-10A	150	300	210	300
MCF-12F	150	300	220	300

## Data Availability

Data is contained within the article and supplementary material.

## Supplementary Materials

The following supporting information can be downloaded at: https://www.mdpi.com/article/10.3390/antiox11030591/s1, Figure S1. Different test concentrations of MCF-7 breast cancer cells treated with (A) 20 µM, 30 µM, 40 µM, and 50 µM CA, (B) 100 µg/mL, 150 µg/mL, 200 µg/mL, and 250 µg/mL CGA, and (C) 40 µM, 80 µM, 120 µM, and 160 µM Arc. Bars represent the mean ± SEM of three biological replicates (*n* = 3); Figure S2. Different test concentrations of MDA-MB-231 breast cancer cells treated with (A) 40 µM, and 45 µM CA, (B) 50 µM CA, (C) 200 µg/mL CGA, (D) 260 µg/mL and 270 µg/mL CGA, (E) 225 µg/mL and 250 µg/mL CGA, and (F) 50 µM, 80 µM, 100 µM, and 150 µM Arc. Bars represent the mean ± SEM of three biological replicates (*n* = 3); Figure S3. Different test concentrations of HCC1419 breast cancer cells treated with (A) 45 µM and 55 µM CA, (B) 250 µg/mL and 275 µg/mL CGA, and (C) 80 µM and 100 µM Arc. Bars represent the mean ± SEM of three biological replicates (*n* = 3); Figure S4. Photomicrographs of MCF-7, MDA-MB-231, and HCC1410 breast cancer and MCF-10A and MCF-12F normal mammary epithelial cells after 4 days of treatments with CA, CGA, and Arc, individually and in combination; Figure S5. Photomicrographs of MCF-7, MDA-MB-231, and HCC1410 breast cancer and MCF-10A and MCF-12F normal mammary epithelial cells on the last day of treatment (Day 7 for MCF-7, MDA-MB-231, MCF-10A and MCF12F cells; Day6 for HCC1419 cells) with CA, CGA, and Arc, individually and in combination; Figure S6. Representative fluorescence microscopy images of (A) MCF-7 and (B) MCF-10A cells treated with CA, CGA, and Arc, alone and in combination for 6 h; Figure S7. (A) OCR, (B) ECAR, and (C) PER rates at baseline, after oligomycin injection, and after rotenone + antimycin A injection in MCF-7 breast cancer cells treated with 35 µM CA, 250 µg/mL CGA, 80 µM Arc, for 48 h; Table S1. Statistical analysis of MCF-7 cell growth under combination treatments. Shown are the *p*-values from posthoc Tukey HSD test following a One-way ANOVA. Differences between treatment groups were considered statistically significant with *p* ≤ 0.05; Table S2. Statistical analysis of MDA-MB-231 cell growth under combination treatments. Shown are *p*-values from post-hoc Tukey HSD test following a One-way ANOVA. Table S3. Statistical analysis of HCC1419 cell growth under combination treatments; Table S4. Statistical analysis of individual and combination treatment comparisons based on ratio of treatment/untreated in MCF-7 cells; Table S5. Statistical analysis of individual and combination treatment comparisons based on ratio of treatment/untreated in MDA-MB-231 cells; Table S6. Statistical analysis of individual and combination treatment comparisons based on ratio of treatment/untreated in HCC1419 cells; Table S7. Statistical analysis of cell numbers of CA, CGA, Arc treatments, alone and in combination, in MCF-10A cells; Table S8. Statistical analysis of cell numbers of CA, CGA, Arc treatments, alone and in combination, in MCF-12F cells.
