# Supplementary material for: Combinatorial Effects of the Natural Products Arctigenin, Chlorogenic Acid, and Cinnamaldehyde Commit Oxidation Assassination on Breast Cancer Cells
Source: Antioxidants (Basel). 2022 Mar 20;11(3):591. doi: 10.3390/antiox11030591 (PMC8945099; doi:10.3390/antiox11030591)
Supplement: Supplementary file 1 [file antioxidants-11-00591-s001.zip › Supplementary Material_manuscript[228].pdf]

SUPPLEMENTARY MATERIAL

Combinatorial Effects of the Natural Products Arctigenin, Chlorogenic Acid, and Cinnamaldehyde Commit Oxidation Assassination on Breast Cancer Cells

Caroline Schuster [1], Nicholas Wolpert [1], Naima Moustaid-Moussa [2,3] and Lauren S. Gollahon [1,3,*]

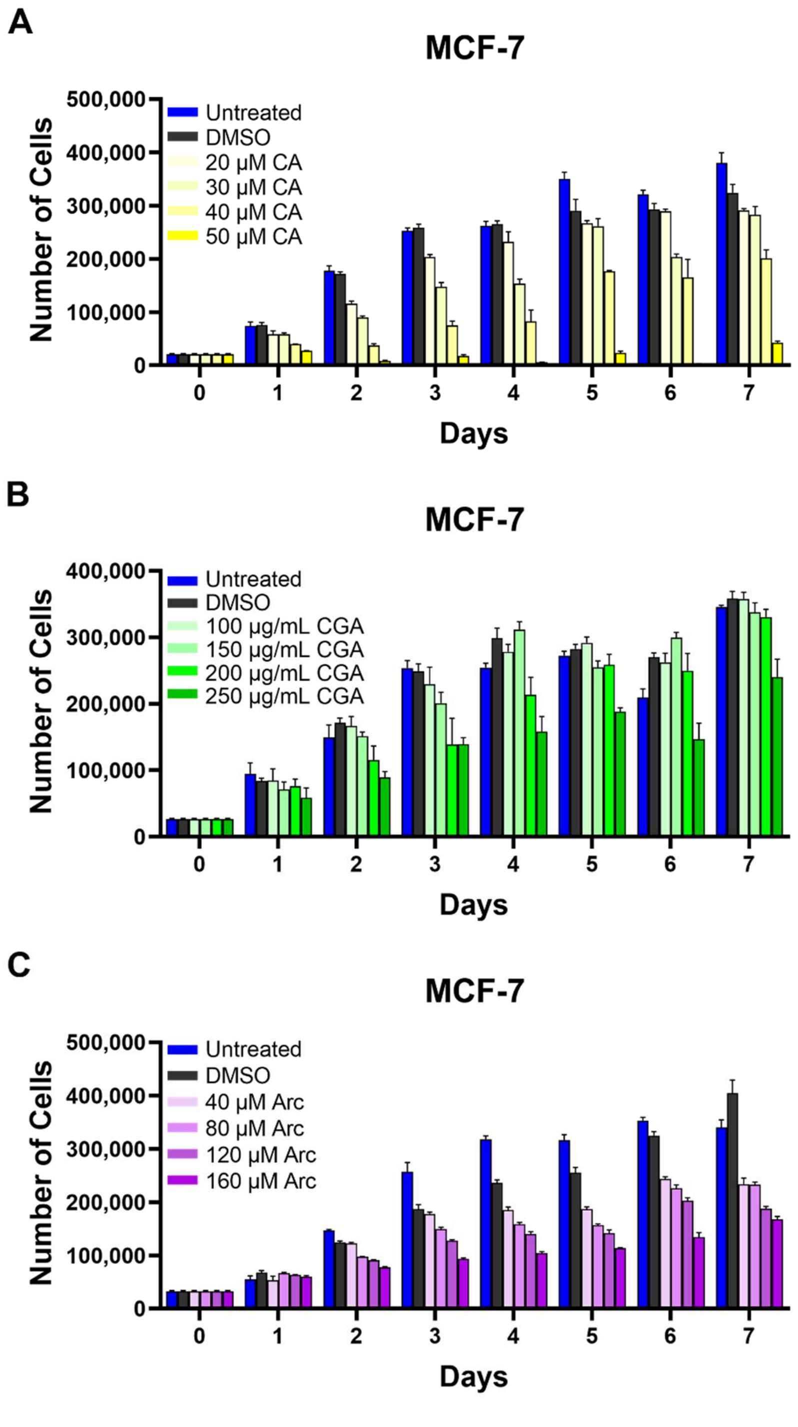

Figure S1. Different test concentrations of MCF-7 breast cancer cells treated with (**A**) 20 µM, 30 µM, 40 µM, and 50 µM CA, (**B**) 100 µg/mL, 150 µg/mL, 200 µg/mL, and 250 µg/mL CGA, and (**C**) 40 µM, 80 µM, 120 µM, and 160 µM Arc. Bars represent the mean ± SEM of three biological replicates (*n*=3).

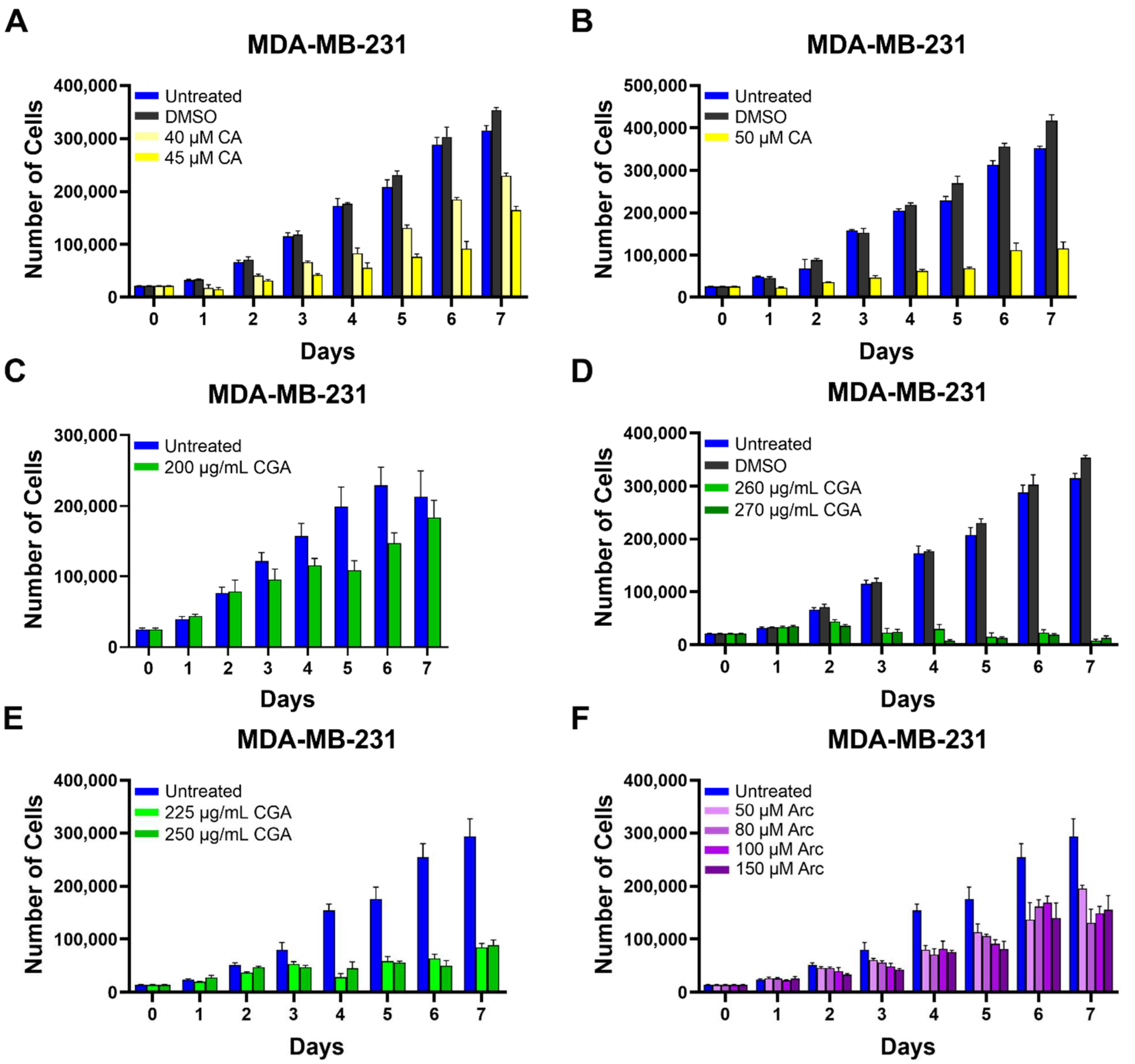

Figure S2. Different test concentrations of MDA-MB-231 breast cancer cells treated with (**A**) 40 μM, and 45 μM CA, (**B**) 50 μM CA, (**C**) 200 μg/mL CGA, (**D**) 260 μg/mL and 270 μg/mL CGA, (**E**) 225 μg/mL and 250 μg/mL CGA, and (**F**) 50 μM, 80 μM, 100 μM, and 150 μM Arc. Bars represent the mean ± SEM of three biological replicates (*n*=3).

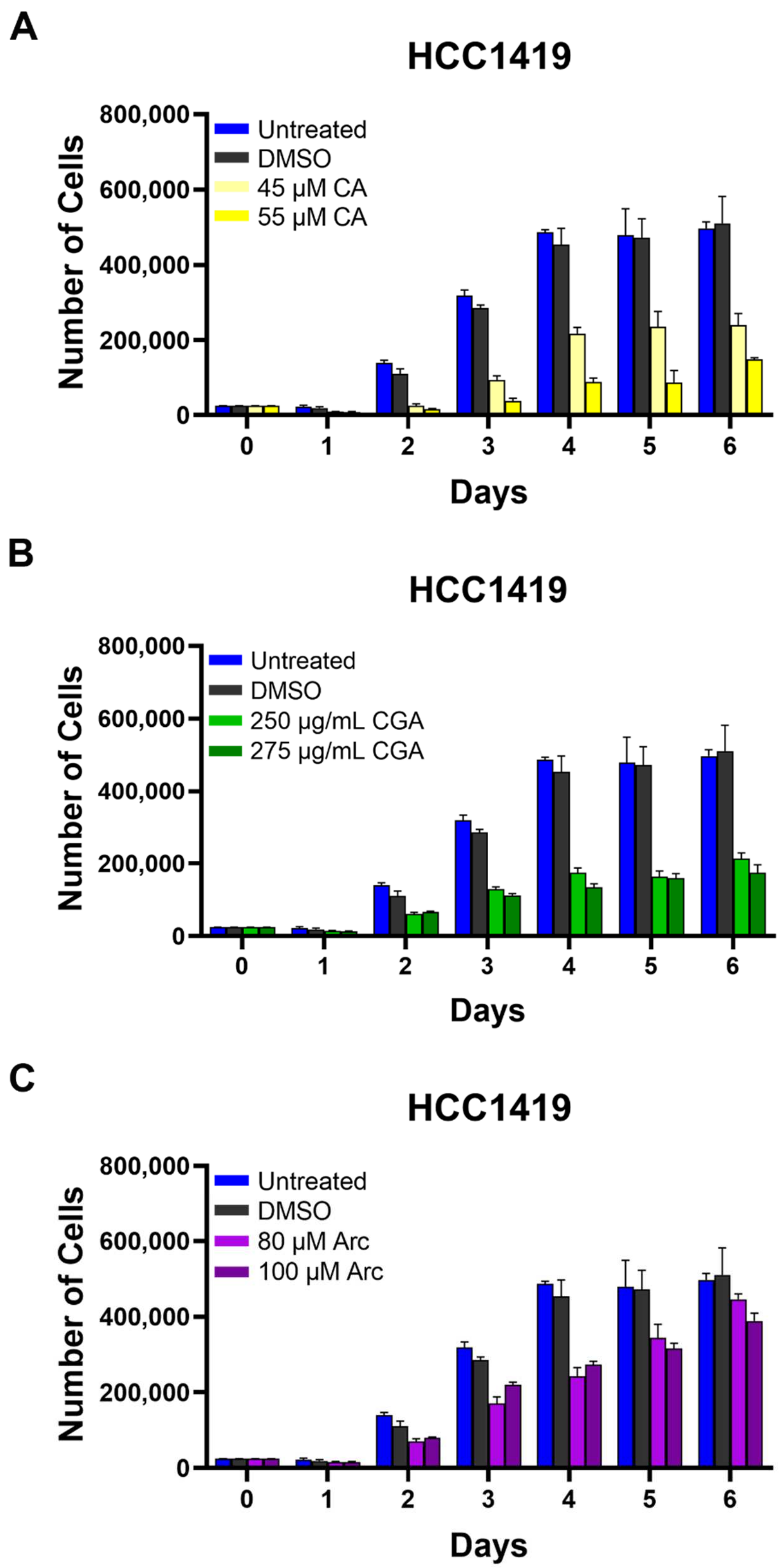

Figure S3. Different test concentrations of HCC1419 breast cancer cells treated with (**A**) 45 µM and 55 µM CA, (**B**) 250 µg/mL and 275 µg/mL CGA, and (**C**) 80 µM and 100 µM Arc. Bars represent the mean ± SEM of three biological replicates (*n*=3).

Table S1. Statistical analysis of MCF-7 cell growth under combination treatments. Shown are the p-values from post-hoc Tukey HSD test following a One-way ANOVA. Differences between treatment groups were considered statistically significant with p≤ 0.05.

Treatment Comparison	Day 1	Day 2	Day 3	Day 4	Day 5	Day 6	Day 7
Untreated - DMSO	0.8370	0.7680	0.0540	0.0000	0.0000	0.1130	0.0930
Untreated - CA+CGA	0.0000	0.0000	0.0000	0.0000	0.0000	0.0000	0.0000
Untreated - CA+Arc	0.0000	0.0000	0.0000	0.0000	0.0000	0.0000	0.0000
Untreated - CGA+Arc	0.4370	0.0000	0.0000	0.0000	0.0000	0.0000	0.0000
Untreated - CA+CGA+Arc	0.0000	0.0000	0.0000	0.0000	0.0000	0.0000	0.0000
DMSO - CA+CGA	0.0000	0.0000	0.0000	0.0000	0.0000	0.0000	0.0000
DMSO - CA+Arc	0.0000	0.0000	0.0000	0.0000	0.0000	0.0000	0.0000
DMSO - CGA+Arc	0.0770	0.0000	0.0000	0.0000	0.0000	0.0000	0.0000
DMSO - CA+CGA+Arc	0.0000	0.0000	0.0000	0.0000	0.0000	0.0000	0.0000
CA+CGA - CA+Arc	0.9220	0.3980	0.7830	0.1490	0.7010	0.9830	0.6090
CA+CGA - CGA+Arc	0.0000	0.0820	0.0070	0.0000	0.0000	0.0040	0.0000
CA+CGA - CA+CGA+Arc	0.6740	1.0000	0.9970	0.9990	0.9710	1.0000	0.9990
CA+Arc - CGA+Arc	0.0010	0.8870	0.0600	0.0000	0.0000	0.0110	0.0010
CA+Arc - CA+CGA+Arc	0.2130	0.3630	0.9530	0.2400	0.9790	0.9700	0.4290
CGA+Arc - CA+CGA+Arc	0.0000	0.0730	0.0140	0.0000	0.0000	0.0030	0.0000

Table S2. Statistical analysis of MDA-MB-231 cell growth under combination treatments. Shown are p-values from post-hoc Tukey HSD test following a One-way ANOVA. Differences between treatment groups were considered statistically significant with $p \leq 0.05$.

Treatment Comparison	Day 1	Day 2	Day 3	Day 4	Day 5	Day 6	Day 7
Untreated - DMSO	0.8618	0.2222	0.9984	0.6056	0.9536	0.6995	0.0497
Untreated - CA+CGA	0.1387	0.0006	0.0000	0.0000	0.0018	0.0000	0.0000
Untreated - CA+Arc	0.0090	0.0001	0.0000	0.0000	0.0119	0.0002	0.0000
Untreated - CGA+Arc	1.0000	0.0058	0.0002	0.0000	0.0099	0.0001	0.0000
Untreated - CA+CGA+Arc	0.0148	0.0000	0.0000	0.0000	0.0014	0.0000	0.0000
DMSO - CA+CGA	0.0215	0.0328	0.0000	0.0000	0.0005	0.0002	0.0000
DMSO - CA+Arc	0.0015	0.0058	0.0000	0.0000	0.0030	0.0013	0.0000
DMSO - CGA+Arc	0.8219	0.2990	0.0001	0.0000	0.0025	0.0009	0.0000
DMSO - CA+CGA+Arc	0.0023	0.0014	0.0000	0.0000	0.0004	0.0001	0.0000
CA+CGA - CA+Arc	0.5883	0.8977	1.0000	0.5536	0.8413	0.7773	0.0085
CA+CGA - CGA+Arc	0.1594	0.7294	0.6097	0.0471	0.8868	0.8814	0.0638
CA+CGA - CA+CGA+Arc	0.7543	0.4315	0.5178	0.9472	1.0000	0.9999	0.9999
CA+Arc - CGA+Arc	0.0104	0.2206	0.5487	0.5619	1.0000	0.9999	0.8287
CA+Arc - CA+CGA+Arc	0.9996	0.9405	0.5782	0.1778	0.7787	0.6705	0.0059
CGA+Arc - CA+CGA+Arc	0.0172	0.0530	0.0487	0.0109	0.8323	0.7939	0.0443

Table S3. Statistical analysis of HCC1419 cell growth under combination treatments. Shown are p-values from post-hoc Tukey HSD test following a One-way ANOVA. Differences between treatment groups were considered statistically significant with $p \leq 0.05$.

Treatment Comparison	Day 1	Day 2	Day 3	Day 4	Day 5	Day 6
Untreated - DMSO	0.9932	0.0126	0.0150	0.1776	0.8162	0.6970
Untreated - CA+CGA	0.0313	0.0000	0.0000	0.0000	0.0000	0.0000
Untreated - CA+Arc	0.0492	0.0000	0.0000	0.0000	0.0000	0.0000
Untreated - CGA+Arc	0.6037	0.0000	0.0000	0.0000	0.0000	0.0000
Untreated - CA+CGA+Arc	0.0198	0.0000	0.0000	0.0000	0.0000	0.0000
DMSO - CA+CGA	0.0126	0.0000	0.0000	0.0000	0.0000	0.0000
DMSO - CA+Arc	0.0198	0.0000	0.0000	0.0000	0.0000	0.0000
DMSO - CGA+Arc	0.3235	0.0000	0.0000	0.0000	0.0000	0.0000
DMSO - CA+CGA+Arc	0.0080	0.0000	0.0000	0.0000	0.0000	0.0000
CA+CGA - CA+Arc	0.9998	0.5202	0.9962	0.9762	0.0103	0.1130
CA+CGA - CGA+Arc	0.3852	0.9574	0.3707	0.7556	0.5449	0.9992
CA+CGA - CA+CGA+Arc	0.9998	0.1207	0.1320	0.1258	0.3196	0.0035
CA+Arc - CGA+Arc	0.5270	0.1741	0.1906	0.9863	0.1776	0.0647
CA+Arc - CA+CGA+Arc	0.9932	0.8886	0.2694	0.0387	0.0003	0.0001
CGA+Arc - CA+CGA+Arc	0.2690	0.0309	0.0044	0.0133	0.0206	0.0061

Table S4. Statistical analysis of individual and combination treatment comparisons based on ratio of treatment/untreated in MCF-7 cells. Shown are p-values from post-hoc Tukey HSD test following a One-way ANOVA. Differences between treatment groups were considered statistically significant with $p \leq 0.05$.

Treatment Comparison	Day 1	Day 2	Day 3	Day 4	Day 5	Day 6	Day 7
CA - CGA	0.9980	0.0950	0.0000	0.9150	0.0000	0.0430	0.9720
CA - Arc	0.8340	0.0210	0.0000	0.8270	0.0000	0.3900	1.0000
CA - CA+CGA	0.4580	0.0190	0.0000	0.0000	0.0000	0.0010	0.0000
CA - CA+Arc	0.7020	0.2350	0.0000	0.0000	0.0000	0.0020	0.0000
CA - CGA+Arc	0.8890	0.6240	0.0010	0.0020	0.0000	0.0320	0.0000
CA - CA+CGA+Arc	0.1810	0.0170	0.0000	0.0000	0.0000	0.0010	0.0000
CGA - Arc	0.5560	0.9770	0.3320	0.2520	0.0000	0.8050	0.9960
CGA - CA+CGA	0.7460	0.0000	0.0000	0.0000	0.0000	0.0000	0.0000
CGA - CA+Arc	0.9280	0.0010	0.0000	0.0000	0.0000	0.0000	0.0000
CGA - CGA+Arc	0.6330	0.0040	0.0000	0.0000	0.0000	0.0000	0.0000
CGA - CA+CGA+Arc	0.3770	0.0000	0.0000	0.0000	0.0000	0.0000	0.0000
Arc - CA+CGA	0.0600	0.0000	0.0000	0.0000	0.0000	0.0000	0.0000
Arc - CA+Arc	0.1250	0.0000	0.0000	0.0010	0.0000	0.0000	0.0000
Arc - CGA+Arc	1.0000	0.0010	0.0000	0.0180	0.0000	0.0010	0.0000
Arc - CA+CGA+Arc	0.0180	0.0000	0.0000	0.0000	0.0000	0.0000	0.0000
CA+CGA - CA+Arc	0.9990	0.7570	0.2940	0.9940	0.9550	1.0000	0.9960
CA+CGA - CGA+Arc	0.0760	0.3290	0.0000	0.3430	0.0000	0.6020	0.1520
CA+CGA - CA+CGA+Arc	0.9930	1.0000	0.9790	1.0000	0.9980	1.0000	1.0000
CA+Arc - CGA-Arc	0.1550	0.9830	0.0010	0.6940	0.0010	0.7360	0.3670
CA+Arc - CA+CGA-Arc	0.9220	0.7280	0.7300	0.9970	0.9990	1.0000	0.9900
CGA+Arc - CA+CGA+Arc	0.0230	0.3060	0.0000	0.3790	0.0000	0.5820	0.1250

Table S5. Statistical analysis of individual and combination treatment comparisons based on ratio of treatment/untreated in MDA-MB-231 cells. Shown are p-values from post-hoc Tukey HSD test following a One-way ANOVA. Differences between treatment groups were considered statistically significant with $p \leq 0.05$.

Treatment Comparison	Day 1	Day 2	Day 3	Day 4	Day 5	Day 6	Day 7
CA - CGA	0.0016	0.8721	0.0137	0.8721	0.0355	0.0014	0.0010
CA - Arc	0.0001	0.5534	0.0667	0.5534	0.9991	0.2014	0.9072
CA - CA+CGA	0.0309	0.4250	0.6072	0.4250	0.0000	0.0000	0.0000
CA - CA+Arc	0.9254	0.0750	0.5258	0.0750	0.0401	0.0003	0.0008
CA - CA+CGA	0.0000	0.9978	0.9997	0.9978	0.0203	0.0002	0.0001
CA - CA+CGA+Arc	0.7169	0.0129	0.0157	0.0129	0.0000	0.0000	0.0000
CGA - Arc	0.7059	0.9958	0.9705	0.9958	0.0156	0.0000	0.0077
CGA - CA+CGA	0.6688	0.0630	0.0006	0.0630	0.0191	0.0319	0.0042
CGA - CA+Arc	0.0113	0.0082	0.0005	0.0082	1.0000	0.9791	1.0000
CGA - CGA+Arc	0.2081	0.5909	0.0273	0.5909	0.9999	0.8348	0.7913
CGA - CA+CGA+Arc	0.0265	0.0014	0.0000	0.0014	0.0085	0.0147	0.0028
Arc - CA+CGA	0.0742	0.0215	0.0029	0.0215	0.0000	0.0000	0.0000
Arc - CA+Arc	0.0007	0.0028	0.0023	0.0028	0.0177	0.0000	0.0059
Arc - CGA+Arc	0.9447	0.2835	0.1270	0.2835	0.0089	0.0000	0.0006
Arc - CA+CGA+Arc	0.0016	0.0005	0.0001	0.0005	0.0000	0.0000	0.0000
CA+CGA - CA+Arc	0.1955	0.9089	1.0000	0.9089	0.0168	0.1328	0.0055
CA+CGA - CGA+Arc	0.0122	0.7281	0.3977	0.7281	0.0334	0.2871	0.0538
CA+CGA - CA+CGA+Arc	0.3826	0.4000	0.2990	0.4000	0.9992	0.9994	1.0000
CA+Arc - CGA-Arc	0.0001	0.1826	0.3305	0.1826	0.9997	0.9985	0.8585
CA+Arc - CA+CGA-Arc	0.9989	0.9511	0.3622	0.9511	0.0075	0.0640	0.0036
CGA+Arc - CA+CGA+Arc	0.0003	0.0341	0.0079	0.0341	0.0149	0.1492	0.0357

Table S6. Statistical analysis of individual and combination treatment comparisons based on ratio of treatment/untreated in HCC1419 cells. Shown are p-values from post-hoc Tukey HSD test following a One-way ANOVA. Differences between treatment groups were considered statistically significant with p≤ 0.05.

Treatment Comparison	Day 1	Day 2	Day 3	Day 4	Day 5	Day 6
CA - CGA	0.1350	0.0004	0.1673	0.0004	0.3029	0.9290
CA - Arc	0.0181	0.0000	0.0004	0.0000	0.0480	0.0000
CA - CA+CGA	1.0000	0.8119	0.0185	0.8119	0.0016	0.1345
CA - CA+Arc	0.9994	0.9387	0.0074	0.9387	0.5661	0.9995
CA - CA+CGA	0.0132	0.2382	0.5717	0.2382	0.0205	0.0696
CA - CA+CGA+Arc	0.9886	0.2440	0.0001	0.2440	0.0001	0.0000
CGA - Arc	0.9052	0.6537	0.0526	0.6537	0.0008	0.0000
CGA - CA+CGA	0.1050	0.0038	0.0002	0.0038	0.1022	0.5816
CGA - CA+Arc	0.2615	0.0001	0.0001	0.0001	0.9983	0.7601
CGA - CGA+Arc	0.8353	0.0291	0.0070	0.0291	0.6863	0.3708
CGA - CA+CGA+Arc	0.0384	0.0000	0.0000	0.0000	0.0042	0.0001
Arc - CA+CGA	0.0138	0.0002	0.0000	0.0002	0.0000	0.0000
Arc - CA+Arc	0.0390	0.0000	0.0000	0.0000	0.0019	0.0000
Arc - CGA+Arc	1.0000	0.0015	0.0000	0.0015	0.0001	0.0000
Arc - CA+CGA+Arc	0.0049	0.0000	0.0000	0.0000	0.0000	0.0000
CA+CGA - CA+Arc	0.9967	0.2711	0.9984	0.2711	0.0419	0.0679
CA+CGA - CGA+Arc	0.0100	0.9130	0.3670	0.9130	0.7897	0.9996
CA+CGA - CA+CGA+Arc	0.9967	0.0239	0.1165	0.0239	0.5850	0.0010
CA+Arc - CGA-Arc	0.0285	0.0425	0.1757	0.0425	0.3996	0.0341
CA+Arc - CA+CGA-Arc	0.9024	0.7758	0.2583	0.7758	0.0017	0.0000
CGA+Arc - CA+CGA+Arc	0.0036	0.0032	0.0024	0.0032	0.0758	0.0019

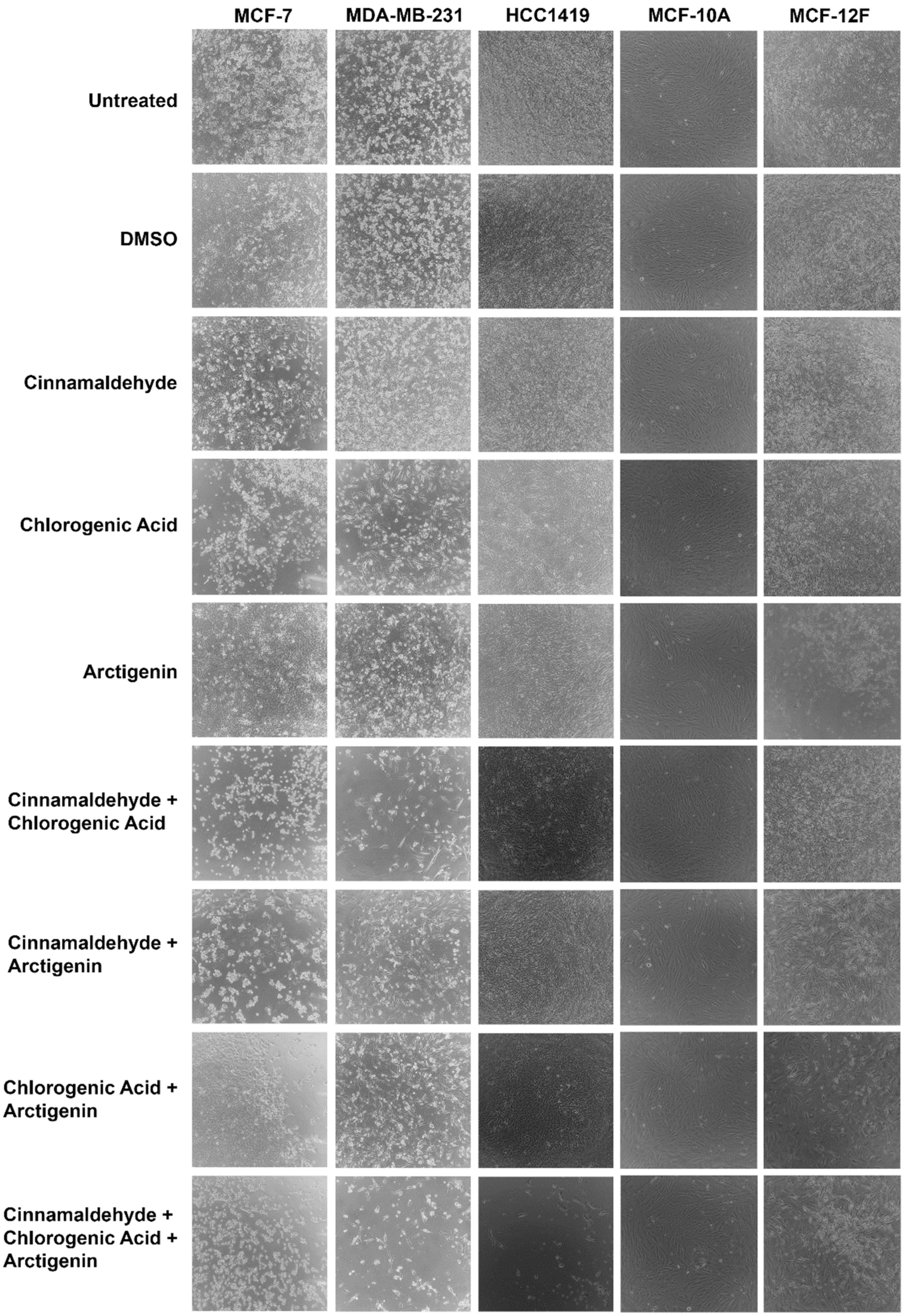

Figure S4. Photomicrographs of MCF-7, MDA-MB-231, and HCC1410 breast cancer and MCF-10A and MCF-12F normal mammary epithelial cells after 4 days of treatments with CA, CGA, and Arc, individually and in combination. Cells were visualized under 10X magnification.

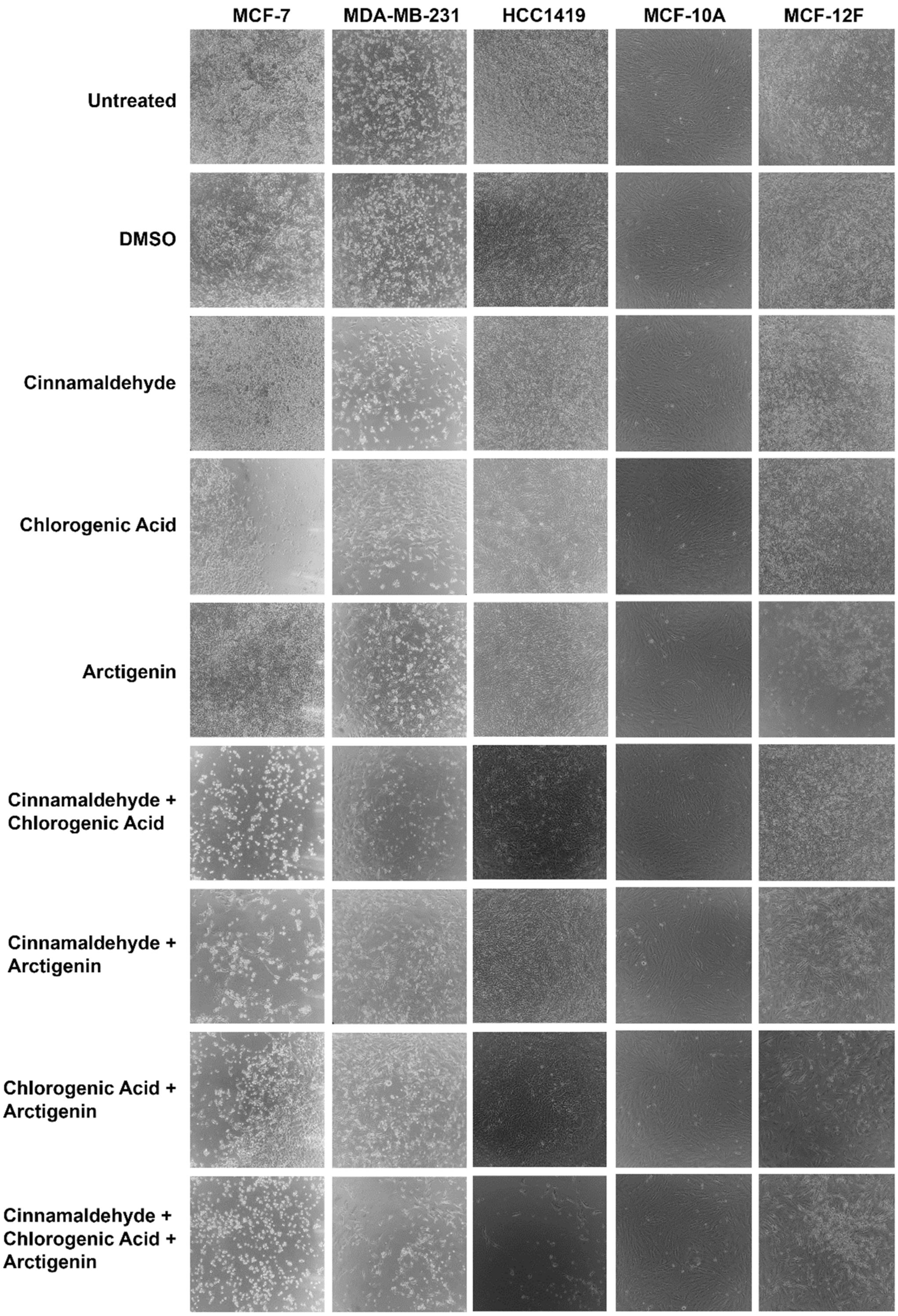

Figure S5. Photomicrographs of MCF-7, MDA-MB-231, and HCC1410 breast cancer and MCF-10A and MCF-12F normal mammary epithelial cells on the last day of treatment (Day 7 for MCF-7, MDA-MB-231, MCF-10A and MCF-12F cells; Day6 for HCC1419 cells) with CA, CGA, and Arc, individually and in combination. Cells were visualized under 10X magnification.

Table S7. Statistical analysis of cell numbers of CA, CGA, Arc treatments, alone and in combination, in MCF-10A cells. Shown are p-values from post-hoc Tukey HSD test following a One-way ANOVA. Differences between treatment groups were considered statistically significant with p≤ 0.05.

Treatment Comparison	Day 1	Day 2	Day 3	Day 4	Day 5	Day 6	Day 7
Untreated - DMSO	0.9297	0.7846	0.0566	0.7527	0.9985	0.9941	0.4829
Untreated - CA	0.9998	0.9352	1.0000	0.9994	1.0000	0.9843	0.5492
Untreated - CGA	0.9998	1.0000	0.0566	0.9134	0.9999	1.0000	1.0000
Untreated - Arc	0.8878	0.7846	0.1191	0.0421	0.0383	0.0024	0.0268
Untreated - CA+CGA	0.8344	1.0000	0.9998	1.0000	0.9999	0.5996	0.9959
Untreated - CA+Arc	1.0000	0.4236	0.0935	0.0017	0.0383	0.0043	0.0420
Untreated - CGA+Arc	0.9798	0.9998	0.0087	0.0014	0.1693	0.0077	0.0027
Untreated - CA+CGA+Arc	1.0000	0.0664	0.0038	0.0003	0.0921	0.0024	0.0214
DMSO - CA	0.9968	0.1644	0.0258	0.9705	0.9996	1.0000	1.0000
DMSO - CGA	0.7003	0.7162	1.0000	1.0000	0.9600	1.0000	0.6169
DMSO - Arc	1.0000	1.0000	1.0000	0.6128	0.1390	0.0136	0.7483
DMSO - CA+CGA	1.0000	0.6430	0.0197	0.9134	1.0000	0.9656	0.9032
DMSO - CA+Arc	0.9600	0.9992	1.0000	0.0532	0.1390	0.0239	0.8596
DMSO - CGA+Arc	1.0000	0.9634	0.9881	0.0421	0.4672	0.0416	0.1800
DMSO - CA+CGA+Arc	0.9297	0.7162	0.9113	0.0098	0.2931	0.0136	0.6840
CA - CGA	0.9798	0.9634	0.0258	0.9978	0.9996	0.9997	0.6840
CA - Arc	0.9912	0.1644	0.0566	0.1304	0.0480	0.0180	0.6840
CA - CA+CGA	0.9798	0.9816	1.0000	1.0000	1.0000	0.9843	0.9375
CA - CA+Arc	1.0000	0.0522	0.0437	0.0060	0.0480	0.0316	0.8074
CA - CA+CGA	0.9998	0.7162	0.0038	0.0047	0.2049	0.0545	0.1485
CA - CA+CGA+Arc	0.9998	0.0054	0.0017	0.0011	0.1134	0.0180	0.6169
CGA - Arc	0.6252	0.7162	1.0000	0.4040	0.0153	0.0058	0.0420
CGA - CA+CGA	0.5490	1.0000	0.0197	0.9854	0.9894	0.8315	0.9996
CGA - CA+Arc	0.9991	0.3584	1.0000	0.0261	0.0153	0.0102	0.0650
CGA - CGA+Arc	0.8344	0.9992	0.9881	0.0205	0.0744	0.0180	0.0042
CGA - CA+CGA+Arc	0.9998	0.0522	0.9113	0.0047	0.0383	0.0058	0.0336
Arc - CA+CGA	1.0000	0.6430	0.0437	0.0841	0.0921	0.1189	0.1217
Arc - CA+Arc	0.9297	0.9992	1.0000	0.8151	1.0000	1.0000	1.0000
Arc - CGA+Arc	1.0000	0.9634	0.9113	0.7527	0.9956	0.9997	0.9627
Arc - CA+CGA+Arc	0.8878	0.7162	0.7280	0.3429	0.9999	1.0000	1.0000
CA+CGA - CA+Arc	0.8878	0.2996	0.0336	0.0036	0.0921	0.1923	0.1800
CA+CGA - CGA+Arc	0.9998	0.9971	0.0029	0.0028	0.3459	0.2979	0.0135
CA+CGA - CA+CGA+Arc	0.8344	0.0409	0.0013	0.0006	0.2049	0.1189	0.0992
CA+Arc - CGA-Arc	0.9912	0.7162	0.9485	1.0000	0.9956	1.0000	0.9032
CA+Arc - CA+CGA-Arc	1.0000	0.9634	0.7994	0.9938	0.9999	1.0000	1.0000
CGA+Arc - CA+CGA+Arc	0.9798	0.1644	1.0000	0.9978	1.0000	0.9997	0.9798

Table S8. Statistical analysis of cell numbers of CA, CGA, Arc treatments, alone and in combination, in MCF-12F cells. Shown are p-values from post-hoc Tukey HSD test following a One-way ANOVA. Differences between treatment groups were considered statistically significant with $p \leq 0.05$.

Treatment Comparison	Day 1	Day 2	Day 3	Day 4	Day 5	Day 6	Day 7
Untreated - DMSO	0.9996	0.0160	0.9976	0.5127	0.6098	0.2571	0.0429
Untreated - CA	0.9996	0.0186	0.9983	0.9994	0.9968	0.9995	1.0000
Untreated - CGA	0.9910	0.2457	1.0000	0.6318	0.8343	0.5256	0.1610
Untreated - Arc	0.9652	0.0004	0.0005	0.0000	0.0000	0.0000	0.0000
Untreated - CA+CGA	0.9962	0.0335	0.9231	0.8688	0.9852	0.9468	0.3880
Untreated - CA+Arc	1.0000	0.0000	0.0002	0.0000	0.0000	0.0000	0.0000
Untreated - CGA+Arc	1.0000	0.0000	0.0001	0.0000	0.0000	0.0000	0.0000
Untreated - CA+CGA+Arc	0.3757	0.0000	0.0000	0.0000	0.0000	0.0000	0.0000
DMSO - CA	0.9652	1.0000	1.0000	0.2230	0.2226	0.0958	0.0429
DMSO - CGA	0.8672	0.8621	0.9822	1.0000	0.0570	0.9998	0.9980
DMSO - Arc	0.7597	0.7143	0.0023	0.0000	0.0000	0.0000	0.0000
DMSO - CA+CGA	1.0000	1.0000	0.9996	0.9990	0.9852	0.0286	0.9259
DMSO - CA+Arc	1.0000	0.0102	0.0008	0.0000	0.0000	0.0000	0.0000
DMSO - CGA+Arc	0.9996	0.0011	0.0004	0.0000	0.0000	0.0000	0.0000
DMSO - CA+CGA+Arc	0.6970	0.0138	0.0002	0.0000	0.0000	0.0000	0.0000
CA - CGA	1.0000	0.8911	0.9856	0.3033	0.9968	0.2384	0.1610
CA - Arc	0.9996	0.6719	0.0021	0.0000	0.0000	0.0000	0.0000
CA - CA+CGA	0.9089	1.0000	0.9994	0.5422	0.7291	0.9992	0.3880
CA - CA+Arc	0.9962	0.0088	0.0008	0.0000	0.0000	0.0000	0.0000
CA - CA+CGA	0.9996	0.0009	0.0004	0.0000	0.0000	0.0000	0.0000
CA - CA+CGA+Arc	0.1581	0.0119	0.0001	0.0000	0.0000	0.0000	0.0000
CGA - Arc	1.0000	0.0897	0.0003	0.0000	0.0000	0.0000	0.0000
CGA - CA+CGA	0.7597	0.9685	0.8184	0.9999	0.3044	0.0801	0.9996
CGA - CA+Arc	0.9652	0.0005	0.0001	0.0000	0.0000	0.0000	0.0000
CGA - CGA+Arc	0.9910	0.0001	0.0001	0.0000	0.0000	0.0000	0.0000
CGA - CA+CGA+Arc	0.0868	0.0007	0.0000	0.0000	0.0000	0.0000	0.0000
Arc - CA+CGA	0.6311	0.4978	0.0073	0.0000	0.0000	0.0000	0.0000
Arc - CA+Arc	0.9089	0.2751	0.9998	0.9222	0.9999	0.9948	0.9259
Arc - CGA+Arc	0.9652	0.0387	0.9928	0.9957	1.0000	0.9948	0.9998
Arc - CA+CGA+Arc	0.0569	0.3409	0.9128	1.0000	0.9143	0.6121	0.9726
CA+CGA - CA+Arc	0.9996	0.0048	0.0026	0.0000	0.0000	0.0000	0.0000
CA+CGA - CGA+Arc	0.9962	0.0005	0.0012	0.0000	0.0000	0.0000	0.0000
CA+CGA - CA+CGA+Arc	0.8170	0.0065	0.0005	0.0000	0.0000	0.0000	0.0000
CA+Arc - CGA-Arc	1.0000	0.9685	1.0000	0.9998	1.0000	1.0000	0.9961
CA+Arc - CA+CGA-Arc	0.4983	1.0000	0.9944	0.9363	0.9927	0.9664	1.0000
CGA+Arc - CA+CGA+Arc	0.3757	0.9374	0.9999	0.9972	0.9797	0.9664	0.9996

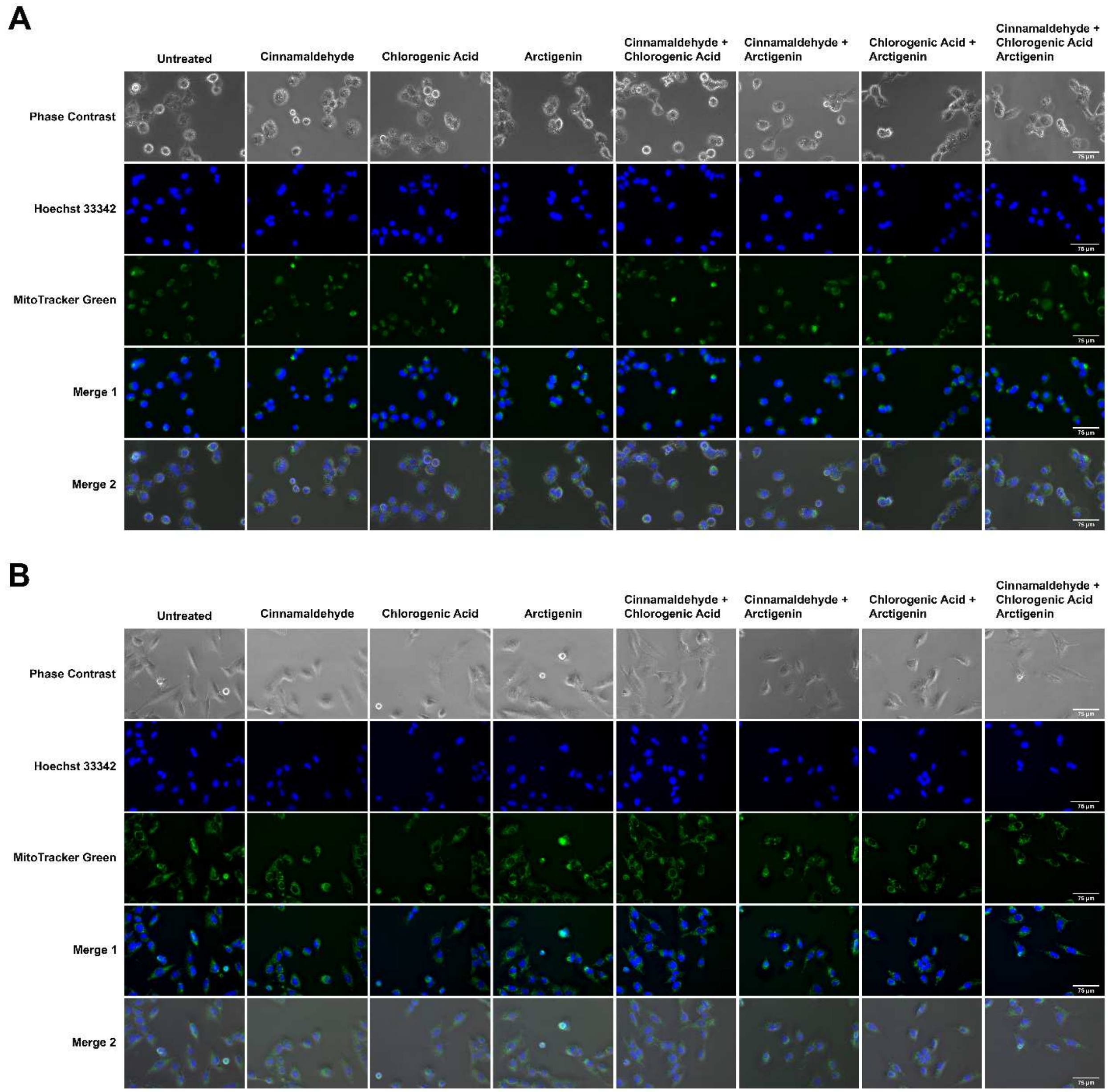

Figure S6. Representative fluorescence microscopy images of (**A**) MCF-7 and (**B**) MCF-10A cells treated with CA, CGA, and Arc, alone and in combination for 6 h. After 6 h treatment, cells were stained with final concentrations of 150 μM MitoTracker® Green FM and 1 μg/mL of Hoechst 33342 before visualization at 40X magnification.

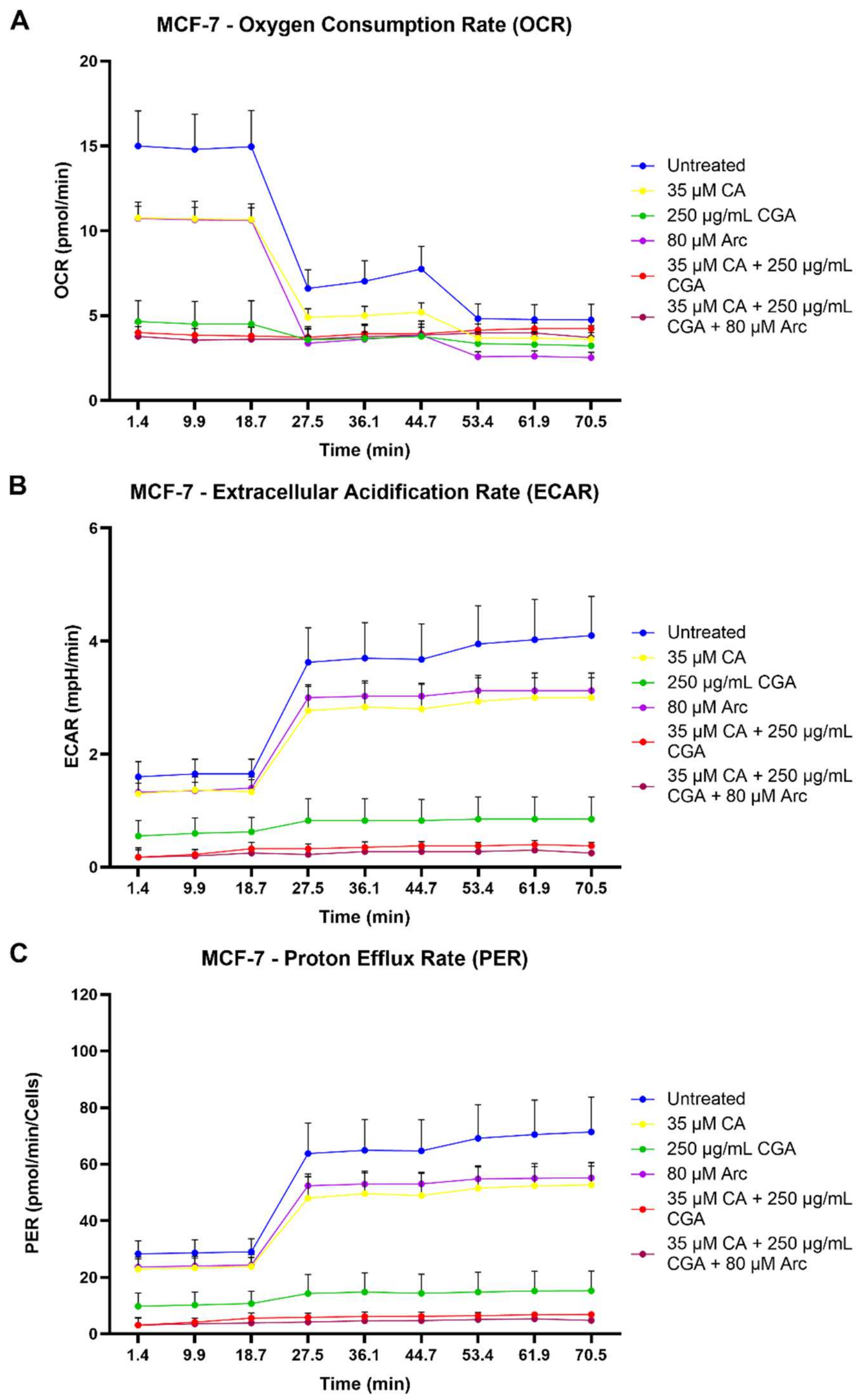

Figure S7. A) OCR, B) ECAR, and C) PER rates at baseline, after oligomycin injection, and after rotenone + antimycin A injection in MCF-7 breast cancer cells treated with 35 μM CA, 250 μg/mL CGA, 80 μM Arc, for 48 h. Points in graphs represent mean of means ± SEM from 3 biological and 4 experimental replicates (*n=4*) of A) OCR (pmol/min), B) ECAR (mpH/min), and C) PER (pmol/min/cells) values in MCF-7 cells of each treatment group. Oligomycin injection occurred at 18.7 min and rotenone A + antimycin A injection at 44.7 min.
